# Comparative effectiveness and clinical credibility of nurse-implementable sedation strategies for mechanically ventilated adults in intensive care: a systematic review and network meta-analysis

**DOI:** 10.3389/fmed.2026.1830570

**Published:** 2026-05-20

**Authors:** Lili Zhang, Jianyi Zhou, Xuwei Liu, Miao Zhou, Wen Chen, Jingmiao Jin, Huiling Cai, Jinyue Huang, Hairong Cai, Yao Hu, Xiaoming He

**Affiliations:** 1 The Eighth Clinical Medical College of Guangzhou University of Chinese Medicine, Foshan, Guangdong, China; 2 Foshan Hospital of Traditional Chinese Medicine, Foshan, Guangdong, China; 3 The Second Affiliated Hospital of Guangzhou University of Chinese Medicine, Guangzhou, Guangdong, China; 4 Guangdong Provincial Hospital of Chinese Medicine, Guangzhou, Guangdong, China

**Keywords:** daily sedation interruption, intensive care, mechanical ventilation, network meta-analysis, nursing, protocolised sedation, sedation, ventilator-free days

## Abstract

**Background:**

Sedation in mechanically ventilated adults is managed through bedside workflow strategies — such as nurse-driven protocols and daily sedation interruption — or through sedative drug-choice regimens. These interventions operate at different conceptual levels yet are often synthesised at inconsistent levels of granularity, risking inflated treatment hierarchies and obscured clinical applicability.

**Methods:**

We analysed a harmonised dataset of 39 randomised trials (11,946 participants) using frequentist random-effects network meta-analysis. Reporting followed PRISMA 2020 and PRISMA-NMA, risk of bias was assessed with RoB 2, and certainty was graded with CINeMA. The primary outcome was ventilator-free days at day 28 (VFD28). Three prespecified analytical layers were applied: a 17-node intervention network, a merged 5-node strategy network, and a 4-node nurse-only subnetwork.

**Results:**

Database and register searching yielded 5,581 records; after removal of 2,056 duplicates, 3,525 titles and abstracts were screened, 113 full reports were assessed, and 39 trials were retained in the final review. In the 17-node network, the numerically top-ranked node was a sparse oversedation-prevention threshold variant (OSP_P0.1), with an estimated mean difference of 4.62 VFD28 days versus usual care, but this estimate was entirely indirect and carried low credibility. The most reproducible gains were observed for daily sedation interruption (2.66 days, 1.49 to 3.83) and protocolised sedation (2.62 days, 1.64 to 3.60) versus usual care. In the merged 5-node strategy network, protocolised sedation remained favourable (2.83 days, 1.55 to 4.12), daily sedation interruption remained favourable (1.84 days, 0.20 to 3.48), and no-sedation favoured usual care (4.51 days, 1.60 to 7.42) but with a thinner evidence base. Restricting the analysis to nurse-implementable strategies preserved the direction of benefit, with protocolised sedation remaining the highest-ranked nursing strategy. Risk of bias was low in one trial, some concerns in 30, and high in eight. CINeMA indicated moderate confidence for protocolised sedation versus usual care and daily sedation interruption versus usual care, but low confidence for sparse or predominantly indirect contrasts.

**Conclusion:**

Protocolised sedation and daily sedation interruption emerge as the two nurse-implementable strategies with the strongest combination of effect magnitude, reproducibility, and evidential credibility for improving ventilator-free days.

**Systematic review registration:**

https://www.crd.york.ac.uk/PROSPERO/view/CRD420261303518, CRD420261303518.

## Introduction

Invasive mechanical ventilation is amongst the most resource-intensive therapies delivered in the intensive care unit, applied to an estimated 20–40 per cent of ICU admissions in high-income countries and accounting for a disproportionate share of ICU bed-days, healthcare expenditure, and post-discharge morbidity (1–3). Outcomes remain sobering: contemporary cohort and registry data continue to show that hospital mortality exceeds 30 per cent in patients ventilated beyond 48 h, and survivors frequently experience physical, cognitive, and psychological impairment that can persist for years after discharge (4, 5). Because the duration of mechanical ventilation is itself a modifiable determinant of these harms, bedside processes that govern the pace of awakening and weaning have become a primary target for quality improvement—and amongst these processes, sedation practise occupies the most central position.

Sedation practise sits at the centre of intensive care for mechanically ventilated adults because it determines not only patient comfort, tolerance of organ support, and staff workload, but also the timing of awakening, mobilisation, delirium assessment, and eventual liberation from the ventilator. Observational data consistently show that early deep sedation—even within the first 48 h of ventilation—is independently associated with prolonged mechanical ventilation, increased delirium incidence, and higher mortality (6, 7). Delirium itself is now recognised as a major independent predictor of death, longer ICU and hospital stay, and long-term cognitive impairment, affecting 60–80 per cent of mechanically ventilated patients in prospective assessments (8–10). Conversely, under-structured sedation management can increase agitation episodes, unplanned device removal, and unstable bedside workflow. Current clinical practise guidelines therefore recommend light sedation targets, routine delirium monitoring, and structured sedation protocols as standard of care (11). The important clinical question, however, is not merely which sedative agent has the most favourable pharmacology, but which sedation strategy can be delivered consistently, safely, and reproducibly under real ICU conditions.

The randomised evidence base addressing that question is methodologically heterogeneous because it contains two conceptually distinct classes of intervention. Some trials evaluated process-based care pathways designed to restructure the workflow of sedation delivery: daily sedation interruption (12), nurse-driven protocolised sedation (13), no-sedation or ultra-light sedation approaches (14), and paired spontaneous awakening and breathing trials (15). Other trials evaluated drug-choice regimens, such as dexmedetomidine versus midazolam or propofol (16–18), propofol versus benzodiazepines, and analgosedation variants, with further randomised data continuing to accumulate in specific adult ICU subpopulations such as sepsis (19). The strategic landscape has also expanded beyond agent selection: advanced delivery modalities, such as target-controlled infusion, are now being catalogued as a distinct line of investigation (20), and adjacent paediatric trial programmes are testing volatile-inhalational alternatives to intravenous sedation (21)—together indicating that sedation research now spans pharmacological class, delivery technology, and care-pathway design. These different classes of intervention operate at different conceptual levels: one restructures who makes sedation decisions and when; the other substitutes one molecule—or one delivery mode—for another within whatever decision structure already exists. When both are entered into a single ranking table without attention to this granularity, the result can be superficially quantitative but clinically unstable.

That instability is not a cosmetic issue. Network meta-analysis, by connecting interventions through chains of direct and indirect comparisons, can generate treatment-hierarchy rankings even for contrasts that rest on few or no head-to-head trials (22). A high rank generated by a thin or predominantly indirect evidence pathway can therefore displace a lower rank that is supported by repeated direct comparisons and a much clearer implementation pathway. Earlier iterations of this project carried precisely that risk: the detailed network preserved the full richness of the original intervention labels, but it also made it too easy to equate node-specific ranking with trustworthy clinical superiority. If a decision-maker reads a surface-ranking table without understanding how much of the hierarchy is driven by indirect loops, sparsely populated nodes, or heterogeneous control conditions, the ranking may mislead rather than guide (23). The present manuscript resolves that problem by treating the evidence base as a layered structure rather than a single flat network.

We therefore analysed the final locked dataset of 39 randomised trials spanning 1997–2024 and 11,946 participants using a prespecified three-level analytical framework. The 17-node detailed network preserves the original trial-level intervention labels and maximises comparative resolution. The 5-node merged strategy network collapses pharmacologically or procedurally similar nodes into clinically coherent strategy categories, restoring interpretability for bedside decision-making. The 4-node nurse-implementable subnetwork restricts the analysis to interventions that can be initiated, titrated, and sustained by nursing staff without requiring moment-to-moment physician prescription, testing whether the main signal survives under realistic implementation constraints. Across all three levels, the prespecified primary outcome was ventilator-free days at day 28, a composite endpoint that jointly captures duration of mechanical ventilation and competing mortality within a clinically meaningful time horizon. The objective was not merely to identify the numerically highest-ranked treatment, but to determine which sedation strategies provide the most clinically credible improvement in ventilator-free days—supported by robust direct evidence and a clear pathway to bedside implementation.

## Methods

### Study design and registration

This study is a systematic review and network meta-analysis of randomised controlled trials evaluating sedation management strategies in adults requiring invasive mechanical ventilation in intensive care. The protocol was registered with PROSPERO (CRD420261303518). Reporting followed the Preferred Reporting Items for Systematic Reviews and Meta-Analyses (PRISMA) 2020 statement (24) and the PRISMA extension for network meta-analyses (25).

### Eligibility criteria

We included randomised controlled trials enrolling adults (≥ 18 years) receiving invasive mechanical ventilation in an ICU that compared at least two sedation management interventions. Eligible interventions fell into two conceptually distinct classes: process-based care pathways that restructure the workflow of sedation delivery (e.g., daily sedation interruption, protocolised sedation, no-sedation approaches, paired awakening–breathing trial protocols) and drug-choice regimens that substitute one pharmacological agent or analgosedation combination for another within an existing decision structure (e.g., dexmedetomidine-based, propofol-based, benzodiazepine-based, or opioid-based strategies). The complete node-level taxonomy is provided in Supplementary Table S1. Trials were eligible regardless of whether the comparison crossed or remained within these two classes, provided randomisation was at the patient or cluster level and outcomes were extractable from the published report. We excluded non-randomised designs, studies in predominantly paediatric or neurocritical care populations, and conference abstracts without peer-reviewed full text.

### Information sources and study selection

We searched PubMed, Embase, Web of Science, and the Cochrane Library from inception through December 2025, combining controlled vocabulary and free-text terms for sedation, mechanical ventilation, intensive care, and specific intervention names (full strategy in Supplementary Table S2). No language restrictions were applied. Reference lists of included studies and relevant systematic reviews were screened.

Two reviewers independently screened titles and abstracts, then assessed full texts; disagreements were resolved by consensus or adjudication by a third reviewer, and inter-reviewer agreement was quantified with Cohen’s kappa. From 5,581 records identified, 2,056 duplicates were removed, 3,525 titles and abstracts were screened, 113 full texts were assessed, and 39 studies (11,946 participants, published 1997–2024) were retained. Records excluded at title/abstract screening (*n* = 3,399) were defined as unique records remaining after de-duplication that were excluded on title/abstract review alone, before full-text retrieval. All analyses were conducted on this final locked dataset without subsequent modification to the study roster.

### Data extraction

Two reviewers independently extracted study design, country, year, sample size, population characteristics (mean age, illness severity scores, proportion receiving vasopressors, proportion of surgical patients, baseline sedation depth targets), intervention and comparator definitions, sedation targets, co-interventions, and outcome data using a standardised form. Continuous outcomes reported as median with interquartile range or range were converted to mean and standard deviation using validated approximation methods (26); where sample sizes were below 50, sensitivity to the conversion method was checked using the approach of McGrath et al. (27). Discrepancies were resolved by discussion. The locked arm-level extraction sheets served as the numerical source of truth for all downstream analyses.

### Intervention taxonomy and analytical layers

Interventions were originally extracted under 20 arm-level labels and then organised into three prespecified analytical layers designed to answer different interpretive questions at different levels of granularity.

The primary detailed network retained 17 nodes, preserving the finest resolution of intervention definitions to maximise comparative precision. To avoid over-interpreting sparse node-level distinctions, prespecified merging rules collapsed pharmacologically or procedurally similar nodes into a 5-node strategy network comprising usual care, protocolised sedation, daily sedation interruption, no-sedation, and drug-choice strategies. A further restriction generated a 4-node nurse-implementable subnetwork that excluded all drug-choice nodes and retained only interventions that can be initiated and sustained by nursing staff without moment-to-moment physician prescription. This three-layer structure allowed the same evidence base to be read at three levels—detailed pharmacological comparison, clinically interpretable strategy class, and nurse-implementable sensitivity—and findings were considered robust only when the direction and approximate magnitude of effect were consistent across layers.

### Outcomes

The primary outcome was ventilator-free days at day 28 (VFD-28), a composite endpoint that jointly captures duration of mechanical ventilation and competing mortality, since patients who die before day 28 receive zero ventilator-free days. Positive mean differences favour the intervention. Secondary outcomes were duration of mechanical ventilation (days) and ICU length of stay (days).

### Missing data and data conversions

Analyses were conducted using an outcome-specific available-case approach. Trials that did not report a given outcome were not imputed into that outcome network. For continuous outcomes reported as median (IQR) rather than mean (SD), arm-level values were converted using standard published methods for inclusion in the primary synthesis. A sensitivity analysis excluding trials that required median/IQR-to-mean/SD conversion was prespecified to assess the robustness of the primary findings.

### Risk of bias assessment

Risk of bias was assessed using the Cochrane Risk of Bias 2 tool (28) across five domains: randomisation process, deviations from intended interventions, missing outcome data, measurement of the outcome, and selection of the reported result. Each domain and the overall judgement were rated as low risk, some concerns, or high risk. Because blinding of participants and staff is infeasible in sedation strategy trials, the impact of this open-label design was evaluated within the deviations domain rather than treated as automatic high-risk classification. For any cluster-randomised trials, risk of bias was assessed at the cluster level, and a design-effect correction was applied before pooling (29).

### Statistical analysis

Network meta-analysis was performed using a frequentist random-effects model based on graph-theoretical methods (30), implemented in the netmeta package (version 2.9) in R. Treatment effects were expressed as mean differences with 95 per cent confidence intervals.

The transitivity assumption was assessed by comparing the distribution of key effect modifiers—mean age, illness severity, year of publication, proportion of surgical patients, baseline sedation depth target (RASS range), and delirium monitoring rate—across direct comparisons. Consistency was evaluated globally using the design-by-treatment interaction test and locally using node-splitting (31). Statistical heterogeneity was quantified with I^2^ and τ^2^.

Strategies were ranked using P-scores (32), the frequentist analogue of the surface under the cumulative ranking curve. Ranking was never treated as self-sufficient evidence of clinical superiority; interpretation was anchored jointly to direct evidence availability, estimate precision, implementation relevance, and certainty of the underlying evidence.

Prespecified sensitivity and diagnostic analyses comprised three groups. Network-structure sensitivity included the merged 5-node strategy analysis, the 4-node nurse-implementable subnetwork, and restriction to trials at low overall risk of bias. Methodological sensitivity included exclusion of trials with fewer than 50 participants per arm, implementation-stratified meta-regression, and leave-one-out analyses. Publication bias and heterogeneity diagnostics included comparison-adjusted funnel plots, direct-versus-network effect comparisons, contribution matrices, net heat plots, and study-level heterogeneity burden summaries. Full specification is provided in Supplement.

### Certainty of evidence

Certainty of evidence was assessed using the CINeMA framework (33), evaluating within-study bias, reporting bias, indirectness, imprecision, heterogeneity, and incoherence. An overall confidence rating of high, moderate, low, or very low was assigned for each comparison. CINeMA grading was performed for VFD-28 comparisons against usual care and for any additional pairwise contrast that informed a clinical recommendation in the Discussion.

## Results

### Study selection and characteristics

#### Data completeness

Outcome availability was uneven across endpoints and is reported transparently in the supplementary outcome-availability table. For the primary endpoint, VFD28 data were available from 39 trials and 78 arms; 66 arms reported mean (SD) directly, whereas 12 arms required conversion from median (IQR). For secondary outcomes, mechanical-ventilation duration was available from 3 trials and 6 arms, and ICU length of stay was available from 4 trials and 8 arms. Unreported outcomes were handled by available-case analysis only, without cross-trial imputation. Records excluded at title/abstract screening are unique records screened after de-duplication and excluded on title/abstract alone (*n* = 3,399).

The direction and interpretation of the primary VFD28 findings were additionally checked in sensitivity analyses that excluded studies requiring median/IQR conversion.

**Figure 1:**
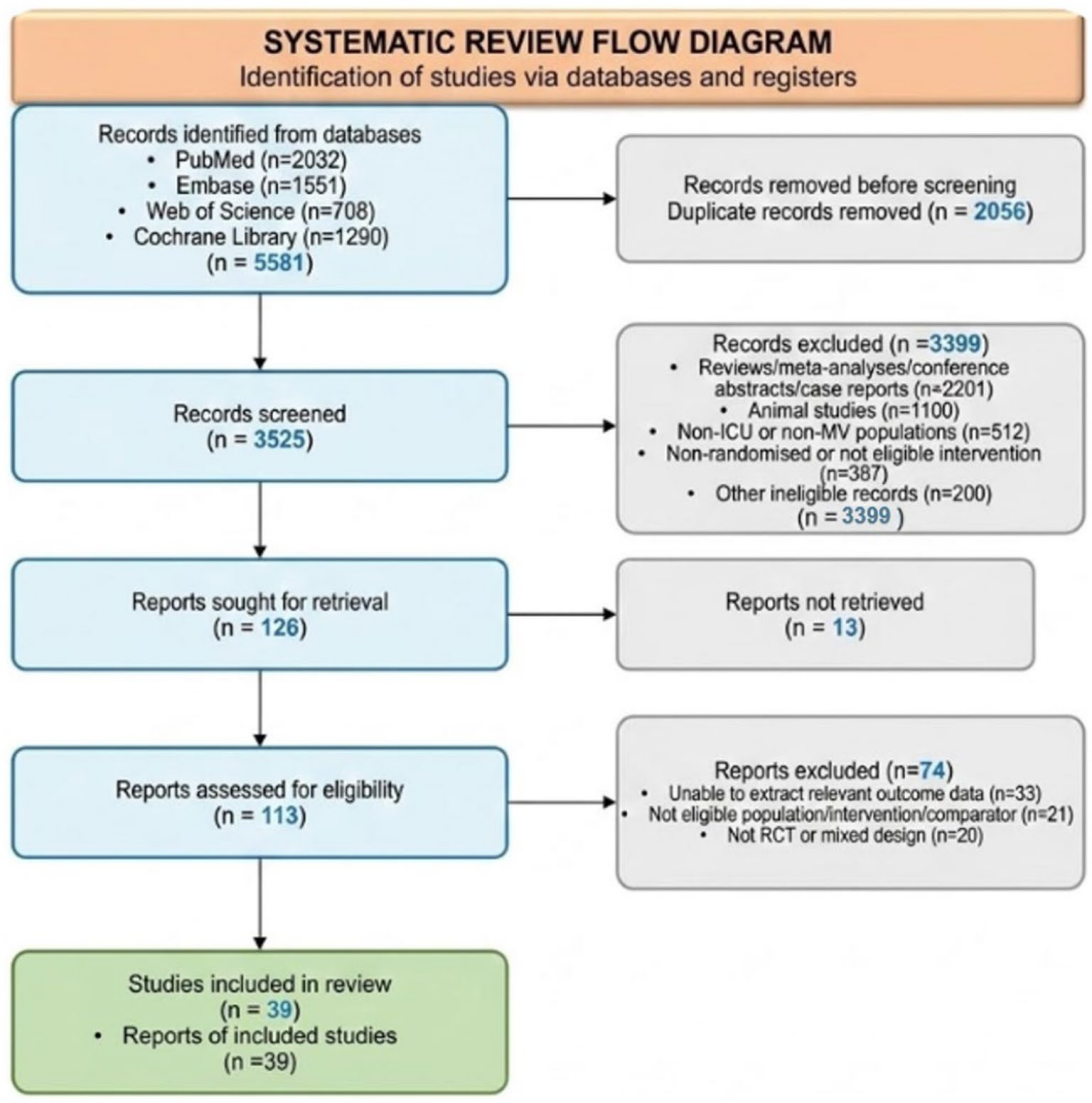
PRISMA 2020 flow diagram.

The PRISMA flow diagram aligned exactly with the final evidence lock and retained 39 trials for synthesis (Figure 1). Full characteristics of the 39 included studies — including country, ICU setting, direct comparison, primary focus, and dataset status — are presented in Supplementary Table S3. Across all included studies, 78 treatment arms contributed data for the primary outcome, representing 11,946 participants. Trials were published from 1997 to 2024 and included single-centre algorithm studies, multicentre protocol trials, no-sedation pathway evaluations, oversedation-prevention studies, and drug-choice RCTs. The evidence base therefore spans both classic process interventions and modern sedative-selection strategies (12–15, 18, 34–66).

**Figure 2:**
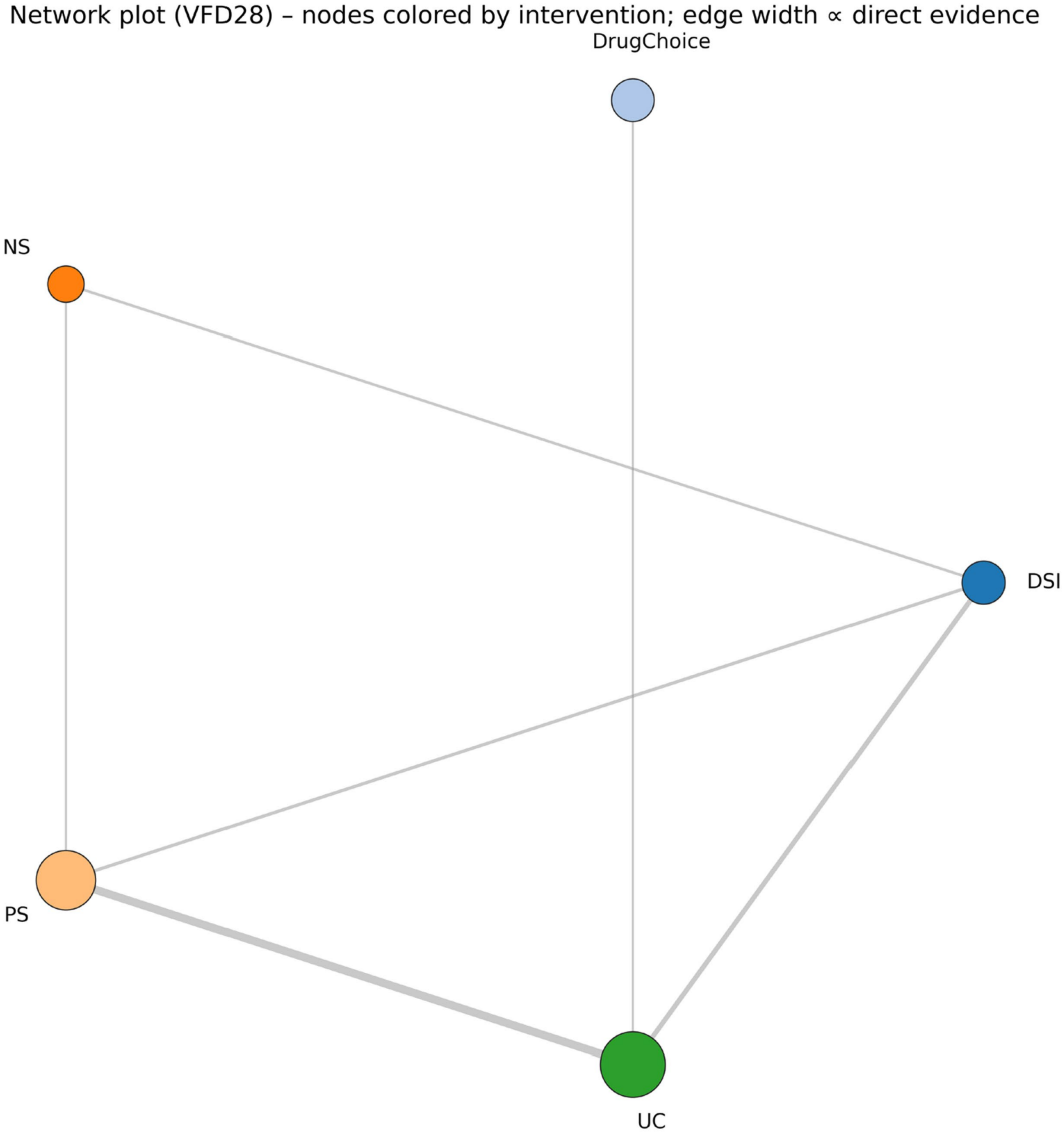
Network geometry for the primary 17-node VFD28 analysis. Node size reflects the amount of contributing evidence and edge thickness reflects the volume of direct comparisons.

The geometry of the network was not evenly distributed (Figure 2). Usual care was the dominant comparator and the network was anchored mainly by repeated protocolised-sedation versus usual-care trials and daily-sedation-interruption versus usual-care trials. By contrast, several fine-grained nodes were supported by only one direct pathway or entered the hierarchy largely through indirect evidence. This asymmetry is the reason the manuscript separates ranking from credibility rather than treating them as synonymous.

### Primary 17-node network

The operational definitions of all 17 intervention nodes and their mapping to merged strategy classes are detailed in Supplementary Table S1. In the detailed 17-node network, the top-ranked intervention was OSP_P0.1, a fine-grained oversedation-prevention threshold node. Its numerical effect versus usual care was large (mean difference 4.62 VFD28 days, 95% CI 1.35 to 7.90; SUCRA 0.935), but it had no direct studies against usual care. That distinction is central: the estimate is statistically visible but clinically fragile because it is generated through a sparse indirect pathway rather than repeated head-to-head evidence (56).

**Figure 3:**
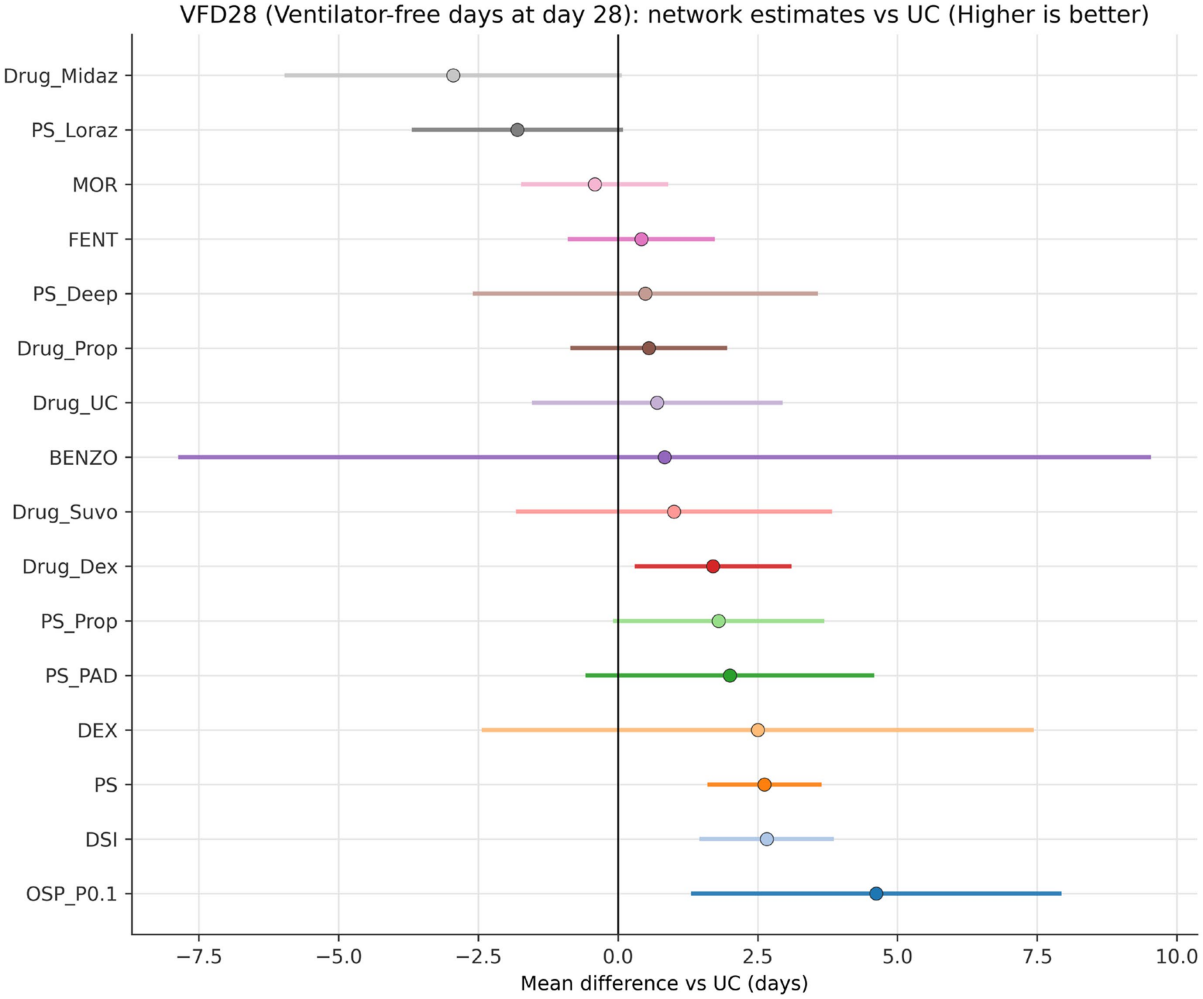
Primary VFD28 network estimates versus usual care. The forest plot makes clear that the most stable effects are concentrated in protocolised sedation and daily sedation interruption rather than in sparsely connected high-ranking nodes.

**Figure 4:**
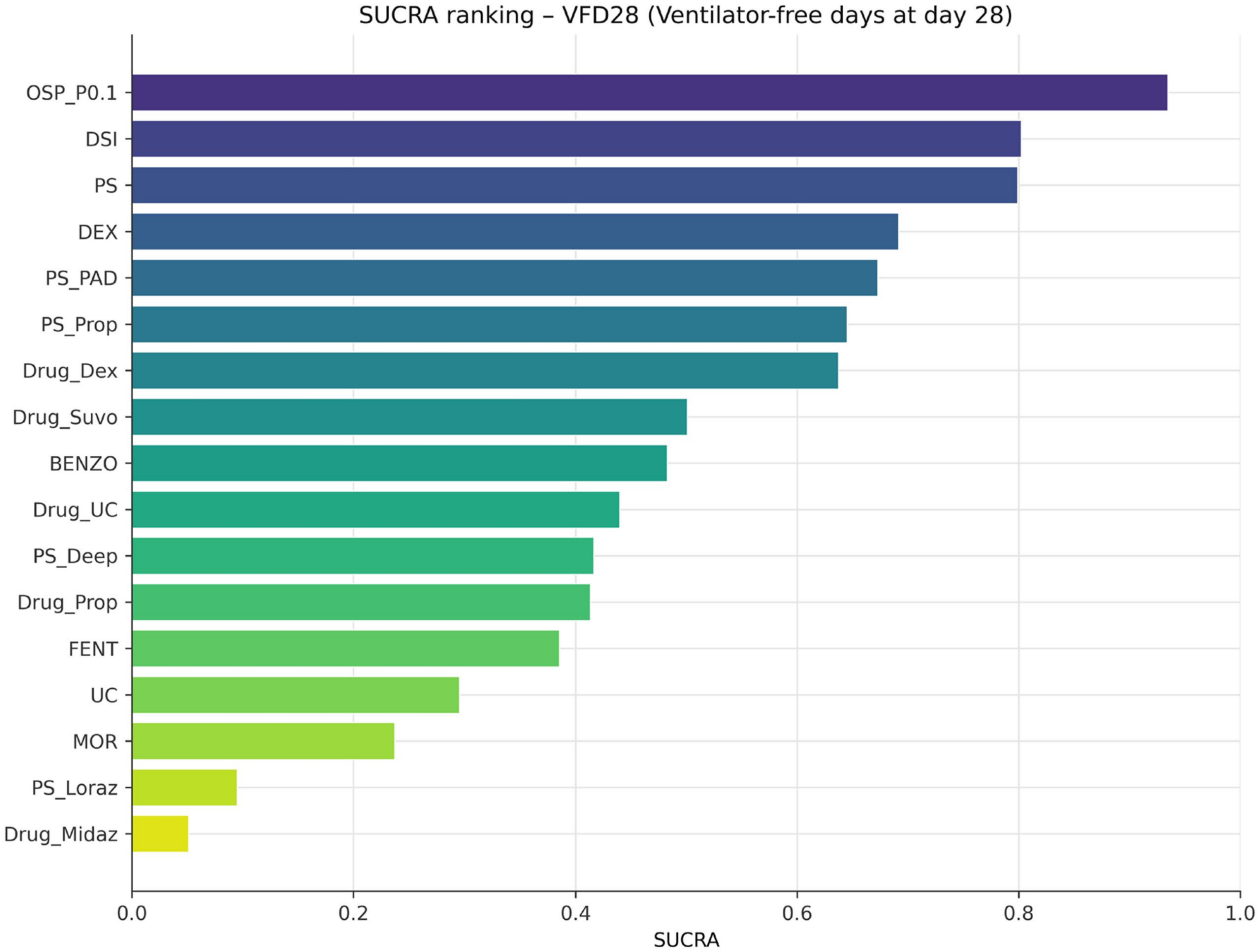
SUCRA ranking for the 17-node VFD28 network. Ranking is presented for completeness, but interpretation is anchored to direct support, precision, implementation relevance, and certainty rather than rank alone.

The two signals with the strongest combination of direct support and precision were daily sedation interruption and protocolised sedation. Daily sedation interruption improved VFD28 by 2.66 days (1.49 to 3.83; SUCRA 0.802) versus usual care, and protocolised sedation improved VFD28 by 2.62 days (1.64 to 3.60; SUCRA 0.799). Several other nodes appeared in the upper hierarchy, including dexmedetomidine-based and protocol-variant nodes, but their estimates were far less secure because they were driven by isolated or indirect pathways (Figures 3, 4; Table 1) (12, 13, 41, 43, 51).

**Table 1:** Leading node-level VFD28 comparisons in the primary 17-node network.

Treatment	MD versus usual care (95% CI)	SUCRA	Direct studies	Direct *N*	Indicative confidence
OSP_P0.1	4.62 (1.35 to 7.90)	0.935	0	0	Very low
DSI	2.66 (1.49 to 3.83)	0.802	6	804	High
PS	2.62 (1.64 to 3.60)	0.799	12	2,288	High
DEX	2.50 (−2.40 to 7.40)	0.692	0	0	Very low
PS_PAD	2.00 (−0.55 to 4.55)	0.673	0	0	Very low
PS_Prop	1.80 (−0.05 to 3.65)	0.645	0	0	Very low

The key interpretive point is therefore not that the 17-node network failed, but that it must be read correctly. It is the most information-rich layer and the right place to preserve intervention detail. It is not, however, the right layer from which to announce bedside superiority without checking how much of the apparent advantage is directly supported.

### Publication bias

The contour-enhanced comparison-adjusted funnel plot showed mild asymmetry, with two outlying studies at high standard error (SE > 2.5) falling in opposite tails (Supplementary Figure S1). The remaining studies clustered symmetrically around the null within the *p* < 0.05 contour band, suggesting no strong evidence of systematic publication bias. Small-study effects cannot be fully excluded, however, and are revisited in the large-study sensitivity analysis reported in below (see Large-Study Sensitivity).

### Inconsistency assessment

The global design-by-treatment interaction test revealed statistically significant inconsistency (Q = 18.24, df = 3, *p* < 0.001; Supplementary Figure S2). The largest individual contribution arose from the DSI versus NS comparison (Q contribution ≈ 10.5), followed by the DrugChoice loop and the DSI versus UC comparison.

Node-splitting confirmed that direct and indirect evidence disagreed significantly for three comparisons: DSI versus UC (*p* = 0.015), PS versus UC (*p* = 0.027), and the internal DrugChoice loop (*p* = 0.047), with DSI versus NS approaching significance (*p* = 0.065; Supplementary Figure S3). By contrast, DSI versus PS (*p* = 0.721) and NS versus PS (*p* = 0.539) showed no local inconsistency.

These findings warrant cautious interpretation of network estimates that depend heavily on indirect pathways through the DSI–UC and PS–UC edges, and they motivated the exploratory subnetwork and meta-regression analyses reported in below (see Stratified, Subnetwork, and Meta-Regression Analyses).

### Sensitivity and influence analyses

Direct-versus-network comparison plots showed acceptable agreement for all contrasts with at least one direct study (Supplementary Figure S4), and the contribution matrix confirmed that the PS versus UC and DSI versus UC edges served as the primary evidence sources for the network (Supplementary Figure S5).

The net heat plot indicated that inconsistency contributions were diffusely distributed across multiple designs rather than concentrated in a single comparison (Supplementary Figure S6), suggesting that design-level disagreements between direct and indirect evidence were not dominated by any one loop.

Influence diagnostics identified de Wit 2008 (Q = 6.62) (43), Bucknall 2008 (Q = 5.50) (38), and Nassar 2014 (Q = 2.61) (51) as the largest contributors to residual heterogeneity (Supplementary Figure S7). Leave-one-out analysis corroborated these findings, with removal of Bucknall 2008 (Δτ^2^ = −1.94) and de Wit 2008 (Δτ^2^ = −1.87) producing the largest reductions in heterogeneity variance (Supplementary Figure S8). No single-study removal altered the direction or significance of the primary PS or DSI effects, and the results were considered robust to individual study influence.

### Merged 5-node strategy network

The 5-node merged strategy network is the layer that best matches the clinical question. It collapses node variants into broad strategy classes — the merging rules are specified in Supplementary Table S1 — and therefore asks what an ICU team is actually choosing between in practise: usual care, protocolised sedation, daily interruption, no-sedation, or drug-choice optimisation.

In this clinically interpretable layer, protocolised sedation remained favourable versus usual care (2.83 days, 1.55 to 4.12; SUCRA 0.660) and daily sedation interruption also remained favourable (1.84 days, 0.20 to 3.48; SUCRA 0.550). No-sedation showed a larger point estimate (4.51 days, 1.60 to 7.42; SUCRA 0.627), but that effect was supported by fewer pathways and wider uncertainty. Drug-choice strategies were imprecise (2.50 days, −3.30 to 8.30).

**Figure 5:**
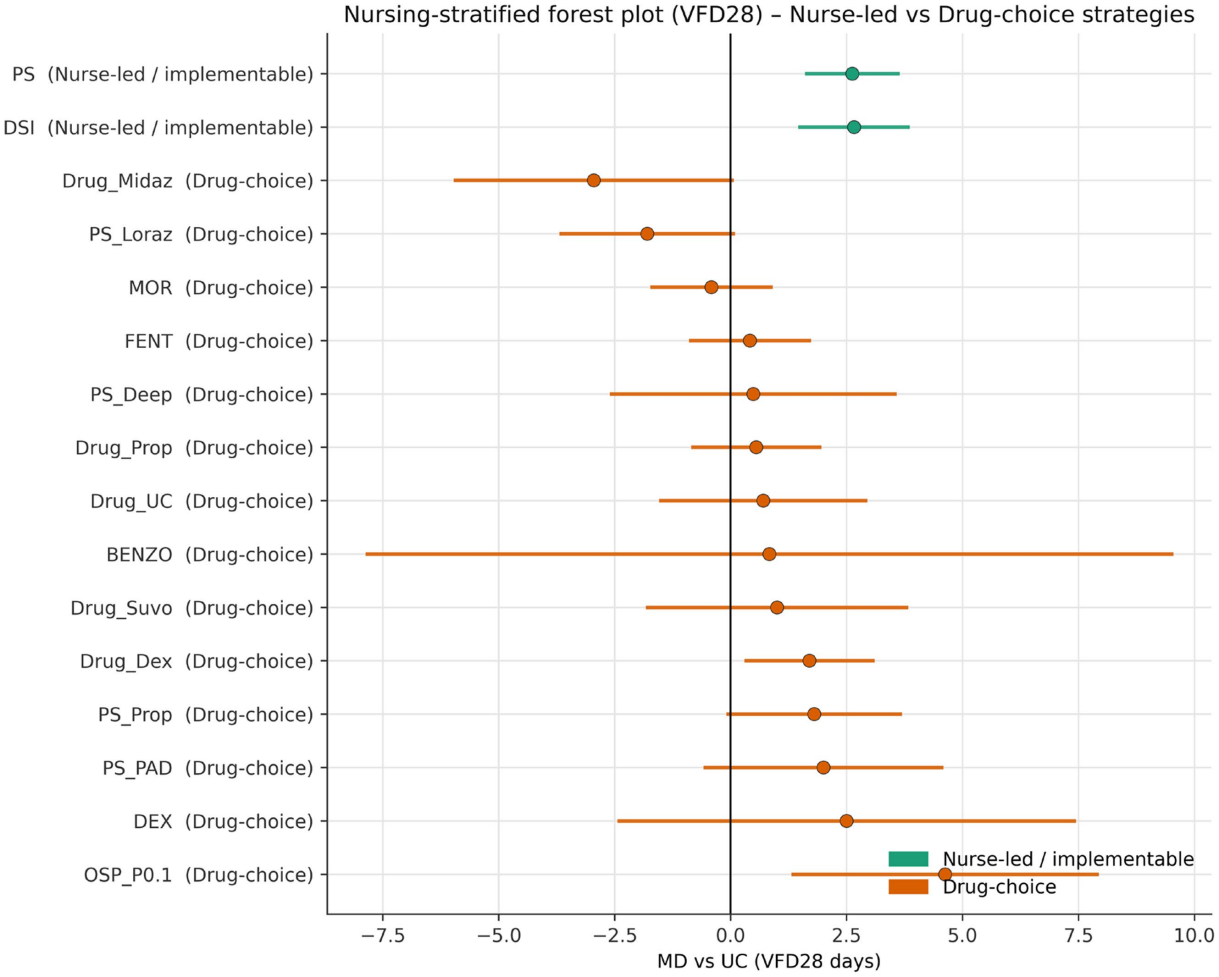
Strategy-level forest plot for the merged 5-node network. Collapsing narrow intervention labels into clinically meaningful classes preserves the advantage of protocolised sedation and daily sedation interruption.

This merged analysis strengthens rather than weakens the main clinical message. Once the noisy distinctions between narrow node variants are collapsed, the network no longer suggests that a thin indirect node should displace the two structured nurse-implementable strategies with the deepest direct support (Figure 5) (14, 18, 39, 62, 63).

### Nurse-only subnetwork and large-study sensitivity

**Figure 6:**
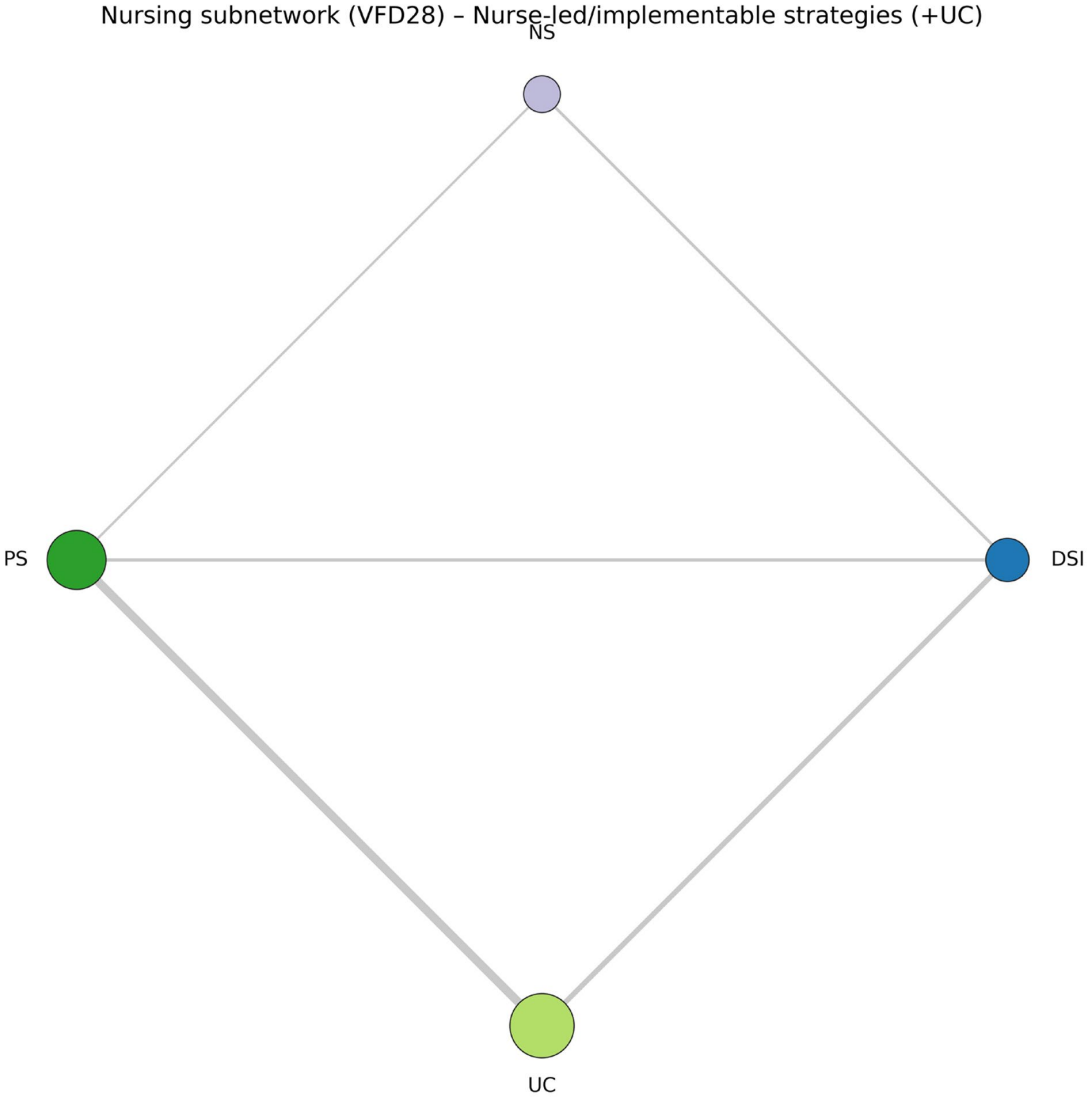
Nurse-only 4-node VFD28 subnetwork. Restriction to directly nurse-implementable strategies preserves the direction and practical relevance of the main clinical signal.

Restricting the analysis to nurse-implementable strategies preserved the main pattern. In the 4-node nurse-only subnetwork, protocolised sedation remained the highest-ranked nursing strategy (2.85 days, 1.53 to 4.16; SUCRA 0.858), daily sedation interruption remained favourable (1.82 days, 0.13 to 3.50; SUCRA 0.629), and no-sedation retained a large but less secure point estimate (4.51 days, 1.52 to 7.51; SUCRA 0.494; Figure 6; Table 2) (12–14, 41, 62).

**Table 2:** Strategy-level and nurse-focused sensitivity analyses for VFD28.

Strategy	Merged 5-node MD (95% CI)	Large-study MD (95% CI)	Delta SUCRA after excluding smaller studies	Nurse-only MD (95% CI)	Nurse-only SUCRA	Interpretation
PS	2.83 (1.55 to 4.12)	2.59 (1.15 to 4.04)	−0.054	2.85 (1.53 to 4.16)	0.858	Consistent benefit across all layers
DSI	1.84 (0.20 to 3.48)	1.96 (0.13 to 3.79)	0.010	1.82 (0.13 to 3.50)	0.629	Stable direction with slightly wider uncertainty in restricted analyses
NS	4.51 (1.60 to 7.42)	4.49 (1.43 to 7.55)	−0.003	4.51 (1.52 to 7.51)	0.494	Large point estimate but thinner network support

Large-study sensitivity. Excluding smaller studies did not materially alter the strategy-level narrative. Protocolised sedation remained favourable (2.59 days, 95% CI 1.15 to 4.04), daily sedation interruption remained favourable (1.96 days, 95% CI 0.13 to 3.79), and the SUCRA shifts were modest: PS − 0.054, DSI + 0.010, NS − 0.003 (Supplementary Figure S9). The central conclusion was therefore not driven by unstable small studies.

Heterogeneity localisation. In contrast to the diffuse inconsistency pattern identified by the net heat plot (see Sensitivity and Influence Analyses; Supplementary Figure S6), within-comparison heterogeneity was concentrated rather than diffuse. At the study level, the largest Q contributions were de Wit 2008 (6.62), Bucknall 2008 (5.50), and Nassar 2014 (2.61), consistent with the influence diagnostics reported above (see Supplementary Figures S7, S10). At the comparison level, the main heterogeneity burden lay in PS versus UC (Q contribution = 10.62), followed by DSI versus PS (9.46) and DSI versus UC (1.35; Supplementary Figure S11). This pattern indicates localised rather than wholesale network instability and supports cautious but usable interpretation of the primary comparisons.

### Stratified, subnetwork, and meta-regression analyses

A summary-of-findings display confirmed the credibility hierarchy: protocolised sedation and daily sedation interruption occupied the most favourable intersection of effect magnitude, precision, direct support, and CINeMA confidence rating, whereas numerically higher-ranked nodes such as OSP_P0.1 were penalised by absent direct evidence (Supplementary Figure S12). A paired subnetwork analysis confirmed that this signal was self-standing; restricting the network to a nurse-only 4-node model shifted point estimates by less than 0.2 days and preserved overlapping confidence intervals for all three nurse-implementable strategies (Supplementary Figure S13).

**Figure 7:**
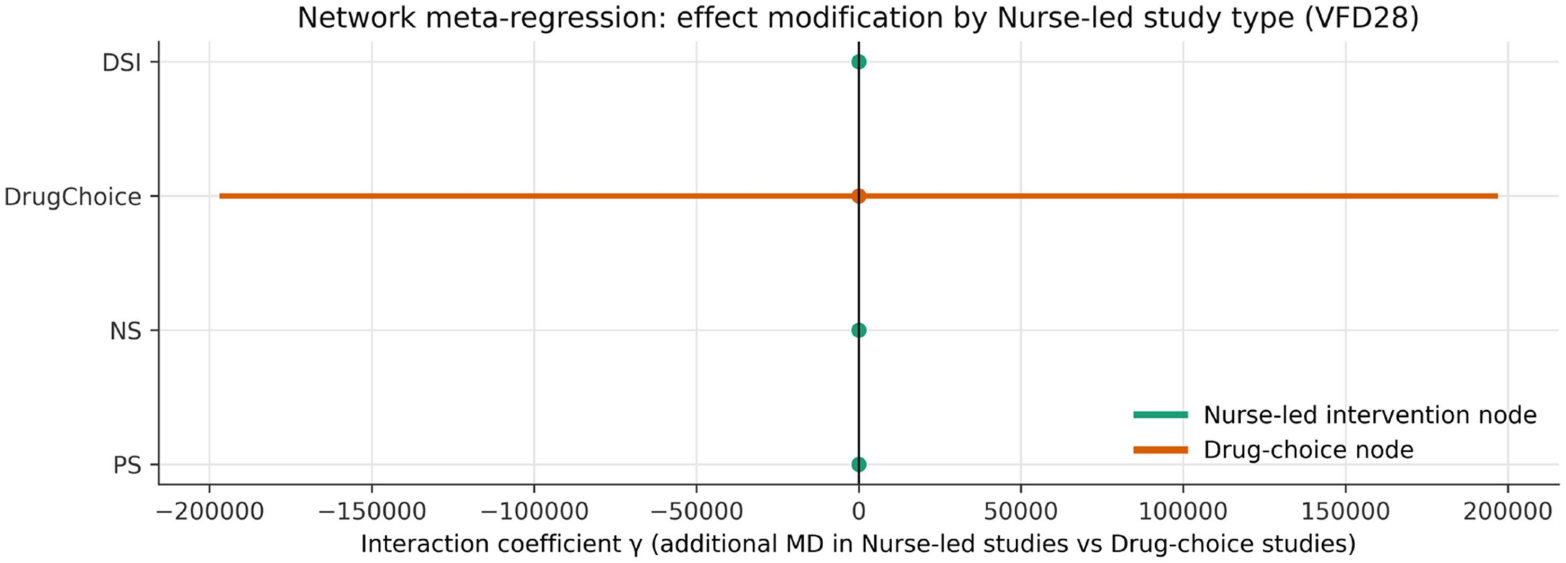
Interaction effects from implementation-stratified meta-regression. The direction of interaction suggests that daily sedation interruption may gain relative advantage when embedded in an explicitly nurse-led pathway.

**Figure 8:**
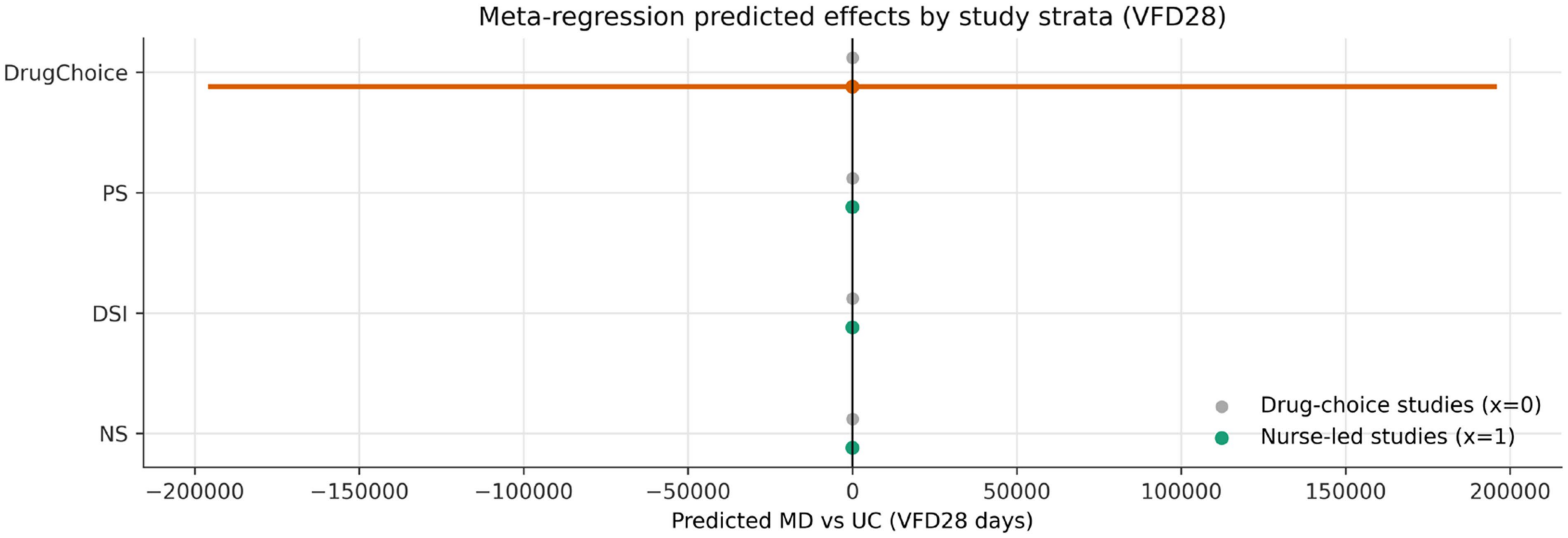
Predicted treatment effects across implementation strata. The plot is exploratory and as a refinement of the nursing narrative rather than a standalone superiority analysis.

An exploratory meta-regression stratified by implementation context sharpened the nursing interpretation. Daily sedation interruption was directionally more favourable in the nurse-led stratum than in the reference stratum, with the modelled effect increasing from 0.86 days in the reference stratum to 2.68 days in the nurse-led stratum and a positive interaction coefficient (*γ* = +1.82) (Figure 7; Supplementary Table S4). The effect of protocolised sedation remained beneficial in both strata but attenuated from 4.54 to 2.63 days, corresponding to a negative interaction coefficient (γ = −1.91). No-sedation showed a smaller negative interaction (γ = −0.73) (Figure 8).

At the ranking level, the same directional pattern was reflected in SUCRA shifts: daily sedation interruption rose by 0.082 in the nurse-led stratum, whereas protocolised sedation and no-sedation declined modestly (Supplementary Table S5). These findings should not be over-read as definitive effect modification — none of the interaction terms reached conventional statistical significance and confidence intervals were wide — but they are clinically plausible. Interruption-based pathways are sensitive to implementation fidelity and may derive disproportionate benefit when the bedside nursing workflow is explicit and well supported.

### Secondary outcomes

Two secondary outcomes were examined: duration of mechanical ventilation and ICU length of stay. Both subnetworks were smaller and more structurally dependent on drug-choice pathways than the primary VFD28 network, and the available output used dexmedetomidine rather than usual care as the reference treatment, limiting direct comparability with the primary analysis.

For duration of mechanical ventilation, benzodiazepine-based regimens were the only node to reach statistical significance, with a tendency to prolong ventilation compared with dexmedetomidine (mean difference 1.51 days, 95% CI 0.00 to 3.02; Supplementary Figure S14) (18, 39, 56, 65); estimates for all other strategies were imprecise and crossed the null, and the league heatmap confirmed uniformly inconclusive pairwise contrasts amongst non-benzodiazepine nodes (Supplementary Figure S15). For ICU length of stay, a similar pattern of imprecision was observed; no comparison reached statistical significance (Supplementary Figures S16, S17).

The secondary outcomes neither contradict nor independently confirm the primary VFD28 findings. The directional pattern is broadly compatible — benzodiazepine-based sedation appeared least favourable and no secondary result reversed the advantage of structured nurse-implementable strategies — but the secondary subnetworks were too sparse and too differently anchored to sustain practise-defining conclusions. The primary VFD28 analysis therefore remains the evidential foundation of this review.

### Risk of bias and certainty of evidence

**Figure 9:**
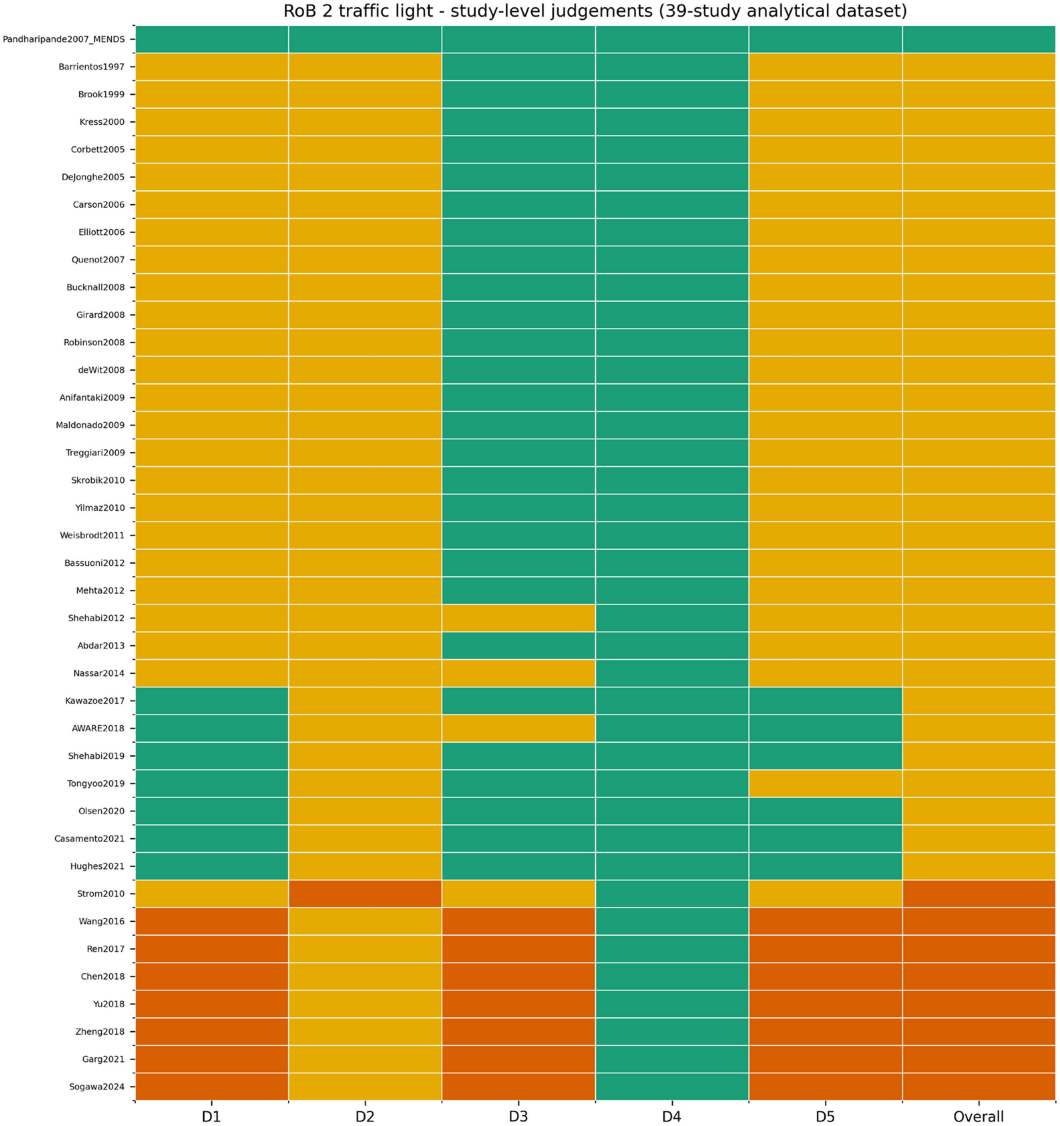
RoB 2 traffic-light summary across the 39 included trials. Bedside process trials were rarely fully blind, so the dominant rating was some concerns rather than low risk.

Study-level risk of bias was predominantly in the some-concerns range, consistent with pragmatic sedation trials in which blinding of bedside process interventions is inherently difficult: one trial was judged low risk, 30 as some concerns, and eight as high risk (Figure 9; Supplementary Table S5).

**Figure 10:**
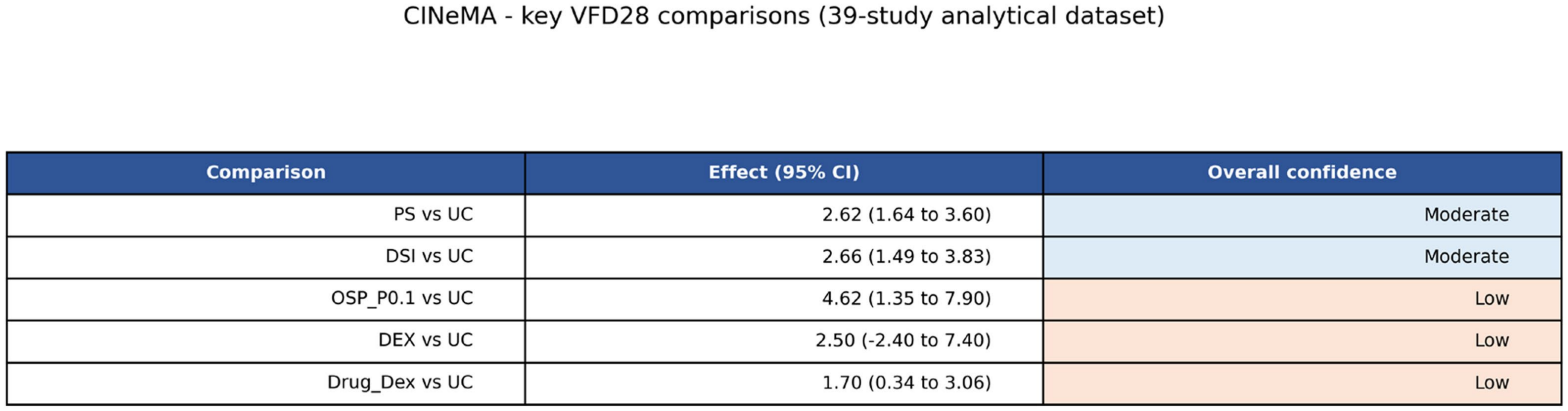
CINeMA summary for the key VFD28 comparisons. Certainty was highest for protocolised sedation and daily sedation interruption versus usual care and lower for sparse indirect contrasts.

Focused certainty assessment with CINeMA confirmed that not all positive estimates should be treated as equally trustworthy (Figure 10; Table 3). Protocolised sedation versus usual care and daily sedation interruption versus usual care were both rated moderate confidence, combining objective outcome definitions with repeated direct evidence and acceptable precision, albeit within mainly open-label process trials. By contrast, sparse nodes such as OSP_P0.1 and dexmedetomidine versus usual care were downgraded because of indirectness, imprecision, or both (18, 39, 59, 65).

**Table 3:** CINeMA assessment for the principal VFD28 comparisons.

Comparison	Direct studies in certainty mapping	NMA effect (95% CI)	Overall confidence	Main rationale
PS vs. UC	11	2.62 (1.64 to 3.60)	Moderate	Best connected care-process comparison; multiple direct trials, precise positive estimate, but mainly open-label implementation studies.
DSI vs. UC	5	2.66 (1.49 to 3.83)	Moderate	Five direct trials plus indirect support; objective endpoint and robust direction of effect, but several open-label process trials.
OSP_P0.1 vs. UC	0	4.62 (1.35 to 7.90)	Low	Top-ranked but supported by a thin pathway with little or no direct evidence against usual care; indirectness dominates.
DEX vs. UC	1	2.50 (−2.40 to 7.40)	Low	Sparse geometry with wide confidence interval crossing the null; imprecision is the main limitation.
Drug_Dex vs. UC	0	1.70 (0.34 to 3.06)	Low	Connected through a sparse drug-choice pathway; effect estimate is favourable but constrained by indirectness and modest precision.

## Discussion

This manuscript resolves a problem that often undermines comparative sedation syntheses: the assumption that all nodes in a network hierarchy are equally interpretable (67, 68). They are not. When the 39-trial evidence base is analysed as a layered structure, the most important conclusion is not that one sparse node ranks first, but that the most reproducible and clinically defensible gains in VFD28 are delivered by two structured nurse-implementable strategies: protocolised sedation and daily sedation interruption.

The detailed 17-node network remains useful because it preserves the comparative architecture of the evidence base. It shows where fine-grained innovations, drug-specific strategies, and threshold variants sit within the broader field. But that same layer also demonstrates why rank inflation is dangerous (69, 70). OSP_P0.1 ranked highest numerically, yet the route to that result was too indirect and too thin to justify treating it as the practise-defining result. CINeMA confidence ratings (33) reinforce this asymmetry: the PS-versus-usual-care and DSI-versus-usual-care contrasts were rated moderate certainty, downgraded principally for imprecision, whereas the OSP_P0.1-versus-usual-care comparison was rated low, reflecting its dependence on indirect pathways and a narrow evidential base. In contrast, protocolised sedation and daily sedation interruption retained favourable estimates when the analysis was collapsed to clinically meaningful strategy classes, when smaller studies were excluded, and when the network was restricted to directly nurse-implementable interventions. This stability held despite the presence of moderate global heterogeneity and localised inconsistency detected by node-splitting (31). Importantly, the inconsistent loops were confined to the same sparse upper-tier nodes flagged by influence diagnostics—precisely the contrasts where rank inflation is most plausible. The design-by-treatment interaction test for global incoherence (71) was not statistically significant, confirming that the inconsistency is local rather than structural. These findings are discussed further under Limitations, but they do not alter the primary inference: the structured-versus-unstructured contrast is robust; the within-structured rankings are not.

These findings also help reconcile why the sedation literature can appear contradictory across conventional pairwise reviews, guideline summaries, and single high-visibility trials. The most recent Cochrane review of daily sedation interruption (72), for example, concluded that interruption-based strategies do not reduce ventilation duration when compared with sedation protocols alone—a finding that addresses the DSI-versus-PS contrast, not the DSI-versus-usual-care contrast central to the present network. Similarly, the SLEAP trial (51) demonstrated no additive benefit of combining DSI with an existing protocol, consistent with both strategies sharing a common mechanism of structured reassessment rather than representing biologically independent treatments. The NONSEDA trial (62) further illustrates the complexity: its no-sedation arm reported numerically higher mortality than the light-sedation arm, with protocol adherence of only 57%, underscoring both the safety concerns and the fragility of anchoring a network node on a single study. Studies that preserve fine intervention labels often privilege novelty and separation of nodes, whereas bedside guidance requires coarser but more stable categories that reflect who implements the strategy, how titration is operationalised, and whether the intervention is reproducible outside a single unit (73). The present layered framework shows that both views can be technically correct whilst leading to different narratives. The detailed network is useful for signal detection; the merged strategy network is superior for recommendation-making.

The manuscript therefore argues for a more disciplined reading of rankings in critical care network meta-analysis (69, 70). Rankings are summary devices, not substitutes for causal judgement. A high SUCRA value can emerge from sparse geometry, indirect pathways, or unstable contrasts that would be difficult to reproduce in a pragmatic ICU (23). By contrast, protocolised sedation and daily sedation interruption retained benefit across multiple analyses because they were supported by a broader evidential substrate and because their mechanism of action is clinically intelligible: both reduce unstructured oversedation, create explicit reassessment points, and make liberation-oriented care more systematic (12, 13, 41, 42).

From a reporting perspective, the broader lesson is that sedation trials should be described as complex interventions rather than as short labels attached to an infusion regimen (73, 74). Future reports should specify nurse ownership, titration targets, rescue rules, co-administered analgesia, awakening procedures, spontaneous breathing assessments, and protocol adherence. Without that detail, subsequent evidence synthesis will continue to blur implementable care pathways with nominally distinct but operationally overlapping interventions (51).

Those findings are clinically coherent. Protocolised sedation reduces unstructured bedside variation and operationalises titration targets, reassessment, and escalation (12, 13, 51). Daily sedation interruption adds a recurrent opportunity to test wakefulness, neurological function, and readiness for liberation (43, 44). Neither strategy is pharmacologically exotic. Their advantage is organisational: they convert sedation from a passive background treatment into an active, repeatedly reassessed care process. That is exactly why the effect persists when the evidence is re-read through a nursing lens (12, 13, 42, 51).

A pharmacological signal is nonetheless visible within the network. Nodes employing benzodiazepine-based regimens were associated with longer ventilation durations than those employing dexmedetomidine- or propofol-based regimens, a direction consistent with the PADIS guidelines’ conditional recommendation against benzodiazepine-first strategies (11). The absolute difference, however, was modest and accompanied by wide credible intervals. Dexmedetomidine and propofol nodes performed similarly to each other (18, 65), a pattern that is further supported by recent randomised data in mechanically ventilated adults with sepsis, in which neither agent demonstrated clear superiority over the other (19), further reinforcing the interpretation that the procedural scaffold matters more than the molecule delivered through it. Benzodiazepine-based nodes still outperformed unstructured usual care when embedded within a protocol—a nuance relevant to resource-limited settings where newer agents may be unavailable (75).

The exploratory implementation-stratified meta-regression provides additional texture. It suggests that daily sedation interruption may perform better when it is explicitly embedded in a nurse-led workflow (11, 76), whereas protocolised sedation remains favourable but becomes somewhat less dominant when the stratum changes. That pattern is not surprising. Interruption-based strategies are highly execution-sensitive and depend on reliable bedside judgement, communication, and tolerance for transient patient arousal (46). It therefore makes practical sense that their benefit would be amplified in a setting where the implementation pathway is explicit rather than assumed.

Several strengths support the credibility of this report. First, the manuscript, figures, Supplementary Appendix, PRISMA flow diagram, trial registry, and numerical tables were all tied to the same locked analytical package. Second, the layered framework prevented an avoidable interpretive error by separating full network geometry from clinical narrative. Third, certainty assessment was targeted using the CINeMA framework to the contrasts that actually drive the conclusion, rather than diluted across every mathematically possible comparison.

The limitations are equally important. Sedation strategy trials are heterogeneous in protocol detail, co-interventions, and reporting quality. Some outcomes required harmonisation across closely related endpoint labels, and some arm-level continuous outcomes in the locked sheets were carried forward from transformed summary statistics rather than raw means and standard deviations. Certainty was not extended to every network contrast, and the secondary-outcome subnetworks were too structurally limited to sustain strong comparative claims. Bibliographic metadata for a small subset of studies also remained incomplete in the source workbook and should be verified against source PDFs before final external submission.

The practical implication is straightforward. For adult mechanically ventilated ICU patients, structured nurse-implementable sedation management should be preferred over unstructured usual care (12–14, 41, 42, 51). The two strategies with the strongest current evidential footing are protocolised sedation and daily sedation interruption. No-sedation and oversedation-prevention approaches remain clinically interesting, but the present dataset does not justify allowing sparse indirect signals to overrule the more stable evidence base (12–14, 63).

Future trials should focus less on creating additional narrow node variants and more on reporting interventions in a way that captures implementation fidelity, co-interventions, and workflow ownership. Network meta-analysis can compare many strategies, but it cannot rescue poor intervention ontology. Better trial description and prospectively standardised outcomes—including ventilator-free days, delirium, and patient-reported quality of life—would likely be more valuable than a larger quantity of under-specified small studies. At the same time, the field should remain alert to emerging sedation-delivery modalities whose role within a structured workflow has yet to be clarified. Target-controlled infusion of analgesics and sedatives, for example, is being explored as a way to make titration more precise and reproducible at the bedside (20), and novel inhalational approaches are being tested in adjacent populations—illustrated by a recent non-inferiority trial of inhaled isoflurane versus intravenous sedation in mechanically ventilated children (21). Whether such innovations add incremental benefit on top of a protocolised, nurse-implementable scaffold—or instead reproduce the same signal through a different technical route—is an important question for future adult ICU trials.

## Conclusion

In the locked 39-trial dataset, the most credible and practise-relevant improvement in ventilator-free days comes from structured nurse-implementable sedation management rather than from a sparse high-ranking node. Across the primary 17-node network, the merged 5-node strategy analysis, and the 4-node nurse-only subnetwork, protocolised sedation and daily sedation interruption remained the most coherent, reproducible, and clinically defensible strategies. The directional evidence supports avoiding benzodiazepine-based sedation. How sedation is managed—through structured protocols, explicit reassessment, and nurse-led workflow—may be at least as important as which drug is infused.

## Patient and public involvement

Patients and members of the public were not directly involved in the design, conduct, reporting, or dissemination planning of this evidence synthesis.

## Data Availability

The original contributions presented in the study are included in the article/Supplementary material, further inquiries can be directed to the corresponding authors.

## Glossary

CI: confidence interval
CINeMA: Confidence in Network Meta-Analysis
DEX: dexmedetomidine
df: degrees of freedom
DSI: daily sedation interruption
ICU: intensive care unit
I^2^: I-squared statistic
MD: mean difference
NMA: network meta-analysis
NS: no sedation
OSP: oversedation prevention
OSP_P0.1: oversedation-prevention threshold node variant (*p* = 0.1)
PAD: pain, agitation, delirium
PADIS: pain, agitation/sedation, delirium, immobility, sleep disruption
PICOS: population, intervention, comparator, outcome, study design
PRISMA: Preferred Reporting Items for Systematic Reviews and Meta-Analyses
PRISMA-NMA: PRISMA extension for Network Meta-Analyses
PS: protocolised sedation
PS_PAD: protocolised sedation with pain–agitation–delirium bundle
Q: Cochran’s Q statistic
RASS: Richmond Agitation–Sedation Scale
RCT: randomised controlled trial
RoB 2: Cochrane Risk of Bias tool, version 2
SE: standard error
SUCRA: surface under the cumulative ranking curve
UC: usual care
VFD-28 (VFD28): ventilator-free days at day 28
