# Supplementary material for: Comparative effectiveness and clinical credibility of nurse-implementable sedation strategies for mechanically ventilated adults in intensive care: a systematic review and network meta-analysis
Source: Front Med (Lausanne). 2026 May 20;13:1830570. doi: 10.3389/fmed.2026.1830570 (PMC13229891; doi:10.3389/fmed.2026.1830570)
Supplement: Supplementary file 1 [file Supplementary_file_1.docx]

Supplementary Appendix: extended methods, diagnostics, and supporting analyses for the 39-trial sedation-strategy network meta-analysis

Authors: [insert full author list and affiliations]

Corresponding author: [insert corresponding author details]

Supplementary overview

The main manuscript keeps the clinical narrative focused on the three-layer VFD28 interpretation. This appendix expands the analytical machinery that supports that narrative, including study-selection detail, node definitions, study characteristics, certainty mapping, heterogeneity localisation, and the complete diagnostic figure set.

Supplementary methods

Search frame and final evidence lock

The harmonised PRISMA diagram in the submission package documents 5,581 records identified from PubMed, Embase, Web of Science, and the Cochrane Library. After removal of 2,056 duplicates, 3,525 records were screened, 126 reports were sought, 113 full texts were assessed, and 39 studies were retained.

Node mapping and intervention ontology

One reason sedation networks are easy to misread is that interventions are reported at incompatible conceptual levels. The source workbooks therefore use an explicit node dictionary that separates the original intervention label, the primary analytical node, and the merged strategy class used for the clinically interpretable analysis.

For example, paired awakening-and-breathing bundles are preserved as SAT_SBT in the detailed network but merged into the daily interruption family for the strategy-level analysis; oversedation-prevention and analgesia-based pathway variants are preserved in the detailed layer but merged into the protocolised-sedation strategy class for the broad clinical comparison.

Outcome harmonisation and continuous-data handling

The primary endpoint was VFD28. Compatible labels such as ventilator-free survival were harmonised when the endpoint was operationally equivalent to a day-28 ventilator-free metric. Continuous outcomes reported as medians with IQRs or related dispersion measures were converted within the locked extraction pipeline and the resulting mean/dispersion values were stored in the arm-level sheets used for synthesis.

Diagnostics and sensitivity analyses

The submission package includes contour-enhanced funnel plots, design-by-treatment inconsistency, node-splitting, direct-versus-network comparisons, contribution matrices, net heat plots, influence diagnostics, leave-one-out heterogeneity analysis, and study-level and comparison-level heterogeneity hotspot summaries.

These diagnostics are not ornamental. Their function is to determine whether the principal narrative is undermined by broad inconsistency, small-study effects, or isolated high-leverage studies. In this dataset the diagnostics support a cautious but usable network: residual uncertainty is localised rather than catastrophic, and the core PS/DSI signal remains intact across the main sensitivity analyses.

Table S1. Node dictionary and strategy-merging rules used for the layered analyses.

| **Node** | **Node name** | **Operational definition** | **Merged strategy** |
| --- | --- | --- | --- |
| UC | Usual care / non-protocolized sedation | Local standard practice without an explicit nurse-driven sedation algorithm or mandated daily awakening. | UC |
| DSI | Daily sedation interruption / SAT | Mandated daily interruption of continuous sedatives or formal spontaneous awakening trial (SAT) protocol. | DSI |
| SAT_SBT | Paired awakening + breathing bundle | Formal paired spontaneous awakening trial + spontaneous breathing trial (ABC / wake-up-and-breathe). | DSI |
| NS | No sedation / ultra-light sedation | Protocol targeting no continuous sedatives (analgesia and boluses as needed), or explicit "no sedation... | NS |
| PS | Nurse-driven sedation protocol / algorithm | Sedation scale (RASS/SAS)–guided titration with explicit algorithm; may include daily goals but not ma... | PS |
| OSP | Oversedation prevention / minimal-sedation guideline | Explicit strategy to prevent oversedation (e.g., minimizing hypnotics), often multi-component but seda... | PS |
| ANALG | Analgesia-based sedation protocol | Analgesia-first sedation strategy (e.g., remifentanil-based) with protocolized minimal hypnotic use. | PS |
| LTGT | Light vs deep sedation target | Randomized depth-of-sedation targets (light vs deep) using sedation scales with explicit targets. | LTGT |
| DEX | Dexmedetomidine-based sedation strategy | Primary sedative is dexmedetomidine; nurse titration to target sedation; adjunct analgesia allowed. | DrugChoice |
| PRO | Propofol-based sedation strategy | Primary sedative is propofol; nurse titration to target sedation. | DrugChoice |
| FENT | Fentanyl-based analgosedation strategy | Continuous fentanyl infusion as primary opioid/sedation component. | DrugChoice |
| MOR | Morphine-based analgosedation strategy | Continuous morphine infusion as primary opioid/sedation component. | DrugChoice |
| DrugChoice | Drug-choice (grouped) | Grouping node used only for merged analyses of drug-choice trials. | DrugChoice |

# Table S2. Full Electronic Search Strategies for All Databases

| Database 1: PubMed (via NLM) | |
| --- | --- |
| #1 | "Respiration, Artificial"[Mesh] OR mechanical ventilation[tiab] OR ventilat*[tiab] |
| #2 | sedation[tiab] OR sedative*[tiab] OR analgosedation[tiab] OR "conscious sedation"[Mesh] |
| #3 | "daily sedation interruption"[tiab] OR spontaneous awakening trial*[tiab] OR SAT[tiab] OR sedation protocol*[tiab] OR nurse-driven[tiab] OR protocolized sedation[tiab] OR no sedation[tiab] OR light sedation[tiab] OR dexmedetomidine[tiab] OR propofol[tiab] OR benzodiazepine*[tiab] OR oversedation[tiab] |
| #4 | randomized controlled trial[pt] OR random*[tiab] |
| #5 | #1 AND #2 AND #3 AND #4 |
| Database 2: Embase (via Elsevier) | |
| #1 | 'artificial ventilation'/exp OR 'mechanical ventilation':ti,ab OR ventilat*:ti,ab |
| #2 | 'sedation'/exp OR sedation:ti,ab OR sedative*:ti,ab OR analgosedation:ti,ab |
| #3 | 'daily sedation interruption':ti,ab OR 'spontaneous awakening trial':ti,ab OR 'sedation protocol':ti,ab OR 'nurse driven':ti,ab OR 'protocolized sedation':ti,ab OR 'no sedation':ti,ab OR 'light sedation':ti,ab OR dexmedetomidine:ti,ab OR propofol:ti,ab OR benzodiazepine*:ti,ab OR oversedation:ti,ab |
| #4 | 'randomized controlled trial'/exp OR random*:ti,ab |
| #5 | #1 AND #2 AND #3 AND #4 |
| Database 3: Cochrane Central Register of Controlled Trials (CENTRAL) | |
| #1 | mechanical NEXT ventilation OR ventilat* |
| #2 | sedation OR sedative* OR analgosedation |
| #3 | "daily sedation interruption" OR "spontaneous awakening trial" OR "sedation protocol" OR nurse-driven OR "no sedation" OR "light sedation" OR dexmedetomidine OR propofol |
| #4 | #1 AND #2 AND #3 |
| Database 4: Web of Science Core Collection | |
| #1 | TS=(mechanical ventilation OR ventilat*) |
| #2 | TS=(sedation OR sedative* OR analgosedation) |
| #3 | TS=("daily sedation interruption" OR "spontaneous awakening trial" OR "sedation protocol" OR nurse-driven OR "protocolized sedation" OR "no sedation" OR "light sedation" OR dexmedetomidine OR propofol OR benzodiazepine* OR oversedation) |
| #4 | TS=(random* OR trial) |
| #5 | #1 AND #2 AND #3 AND #4 |

Table S3. Characteristics of the 39 included studies in the locked analytical dataset.

| **Study** | **Country** | **ICU setting** | **Direct comparison** | **Primary focus** | **Dataset status** |
| --- | --- | --- | --- | --- | --- |
| Barrientos 1997 | Spain | NR | Drug_Midaz; Drug_Prop | Sedation management strategy | Extracted |
| Brook 1999 | NR | NR | PS; UC | PS vs UC | ToRetrieve |
| Kress 2000 | NR | NR | DSI; UC | DSI vs UC | ToRetrieve |
| Corbett 2005 | USA | NR | Drug_Dex; Drug_Prop | Sedation management strategy | Extracted |
| De Jonghe 2005 | France | NR | PS; UC | Sedation management strategy | Extracted |
| Carson 2006 | NR | NR | PS_Loraz; PS_Prop |  | TO_EXTRACT |
| Elliott 2006 | Australia | NR | PS; UC | Sedation management strategy | Extracted |
| Pandharipande 2007 | NR | NR | BENZO; DEX | DEX vs LOR | ToRetrieve |
| Quenot 2007 | France | NR | PS; UC | Sedation management strategy | Extracted |
| Bucknall 2008 | NR | NR | PS; UC | PS vs UC | ToRetrieve |
| Girard 2008 | USA | Mixed ICUs (4 ICUs) | SAT_SBT; UC | Paired SAT + SBT protocol vs SBT with usual care sedation | Extracted |
| Robinson 2008 | USA | NR | PS; UC | Sedation management strategy | Extracted |
| de Wit 2008 | USA | Medical ICU | DSI; PS | Daily interruption of sedation vs nursing-implemented s... | Extracted |
| Anifantaki 2009 | NR | NR | DSI; UC | DSI vs UC | ToRetrieve |
| Maldonado 2009 | USA | NR | Drug_Dex; Drug_Prop | Sedation management strategy | Extracted |
| Treggiari 2009 | USA | NR | PS; PS_Deep | Sedation management strategy | Extracted |
| Skrobik 2010 | Canada | NR | PS_PAD; UC | Sedation management strategy | Extracted |
| Strøm 2010 | Denmark | General ICU | DSI; NS | No sedation vs sedation+DSI | Extracted |
| Yilmaz 2010 | NR | NR | DSI; UC |  | TO_EXTRACT |
| Weisbrodt 2011 | NR | NR | DSI; UC | DSI vs UC | ToRetrieve |
| Bassuoni 2012 | Egypt | NR | DSI; NS | Sedation management strategy | Extracted |
| Mehta 2012 | NR | NR | DSI; PS | DSI + PS vs PS | ToRetrieve |
| Shehabi 2012 | Aus/NZ | NR | PS; PS_Deep | Sedation management strategy | Extracted |
| Abdar 2013 | Iran | NR | PS; UC | Sedation management strategy | Extracted |
| Nassar Jr. 2014 | Brazil | ICU | DSI; PS | Intermittent sedation vs DSI | Extracted |
| Wang 2016 | China | NR | PS; UC | Sedation management strategy | Extracted |
| Kawazoe 2017 | Japan | ICU (sepsis) | DEX; UC | Dexmedetomidine adjunct vs no dex (light sedation) | Extracted (abstract-level) |
| Ren 2017 | China | NR | PS; UC | Sedation management strategy | Extracted |
| Chen 2018 | China | NR | DSI; UC | Sedation management strategy | Extracted |
| De Jonghe 2018 | France | Multicenter ICU | OSP; UC | Oversedation prevention vs control | Extracted |
| Yu 2018 | China | NR | PS; UC | Sedation management strategy | Extracted |
| Zheng 2018 | China | NR | PS; UC | Sedation management strategy | Extracted |
| Shehabi 2019 | Multi | NR | Drug_Dex; Drug_UC | Sedation management strategy | Extracted |
| Tongyoo 2019 | Thailand | NR | OSP_P0.1; PS | Sedation management strategy | Extracted |
| Olsen 2020 | Scand | NR | NS; PS | Sedation management strategy | Extracted |
| Casamento 2021 | Australia | ICU | FENT; MOR | Fentanyl vs morphine infusion (analgosedation choice) | Candidate (needs arm Ns/full text) |
| Garg 2021 | India | NR | Drug_Dex; Drug_Prop | Sedation management strategy | Extracted |
| Hughes 2021 | USA | Multicenter ICU (sepsis) | Drug_Dex; Drug_Prop | Dexmedetomidine vs propofol (light sedation; nurse titr... | Candidate (needs unadjusted distribution) |
| Sogawa 2024 | Japan | NR | Drug_Suvo; UC | Sedation management strategy | Extracted |

Table S4. Meta-regression directionality and heterogeneity highlights.

| **Strategy** | **Interaction gamma** | **Delta SUCRA in nurse-led stratum** | **Interpretation** |
| --- | --- | --- | --- |
| DSI | 1.82 | 0.082 | Directionally strengthened in nurse-led stratum |
| PS | -1.91 | -0.060 | Remained beneficial but attenuated |
| NS | -0.73 | -0.086 | Negative interaction with modest rank loss |

Table S5. Overall RoB 2 distribution across included trials.

| **Overall RoB** | **n** | **%** |
| --- | --- | --- |
| Low | 1 | 2.6% |
| Some concerns | 30 | 76.9% |
| High | 8 | 20.5% |


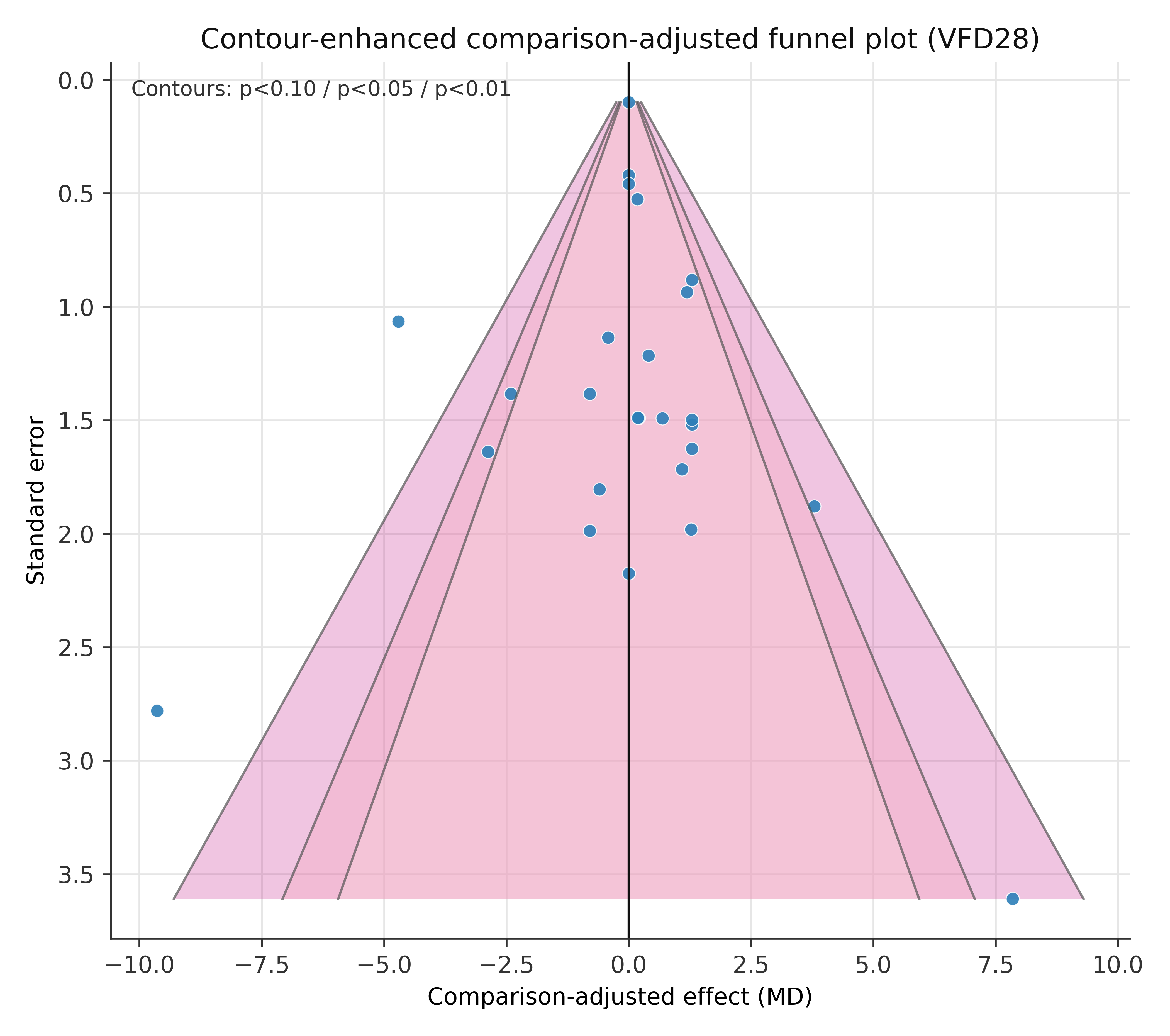


Fig. S1. Contour-enhanced funnel plot for the primary VFD28 network. This is presented as a small-study-effects screen.


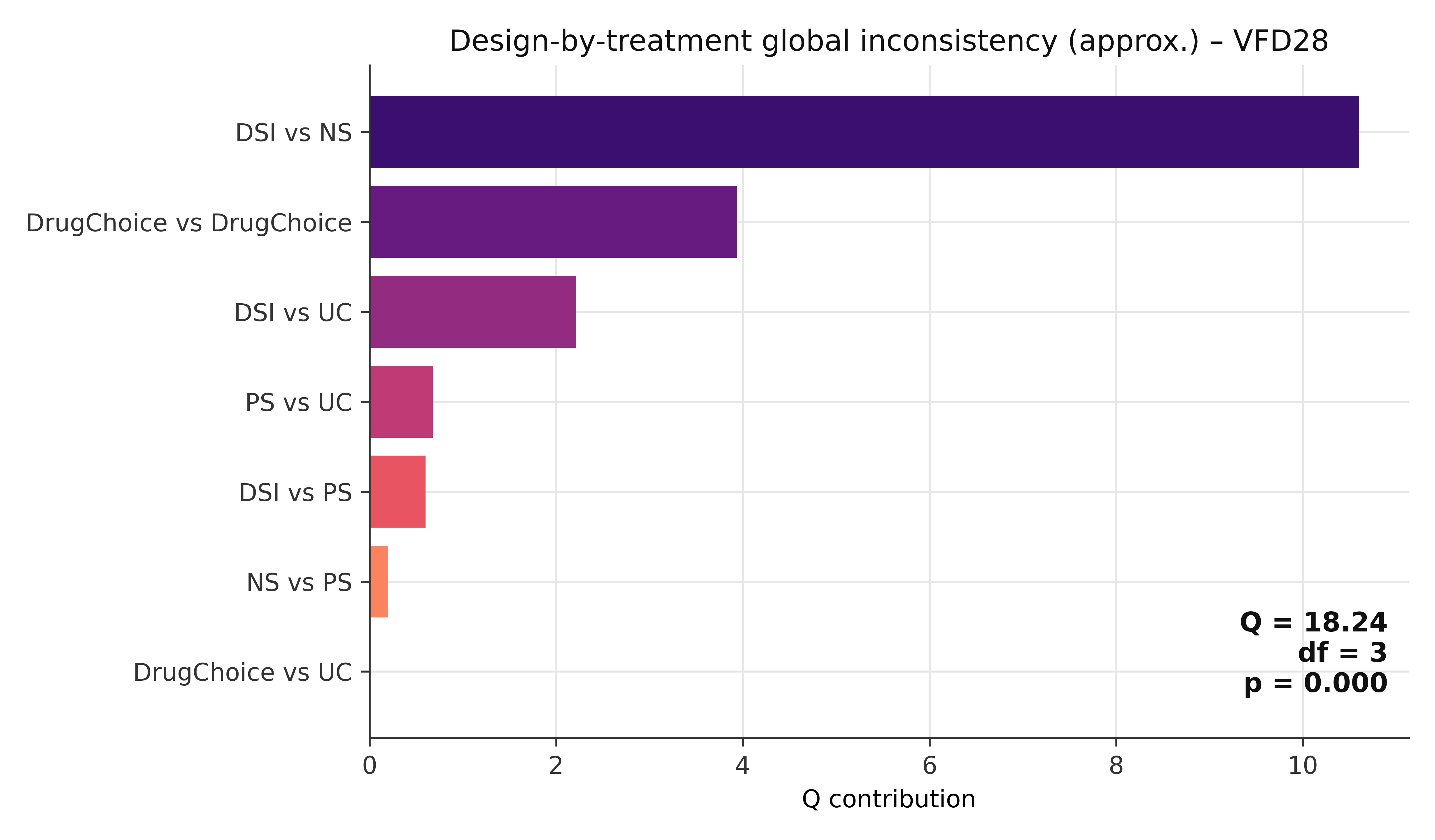


Fig. S2. Design-by-treatment global inconsistency assessment. The purpose of this figure is to detect network-wide inconsistency that would undermine the main comparative framework.


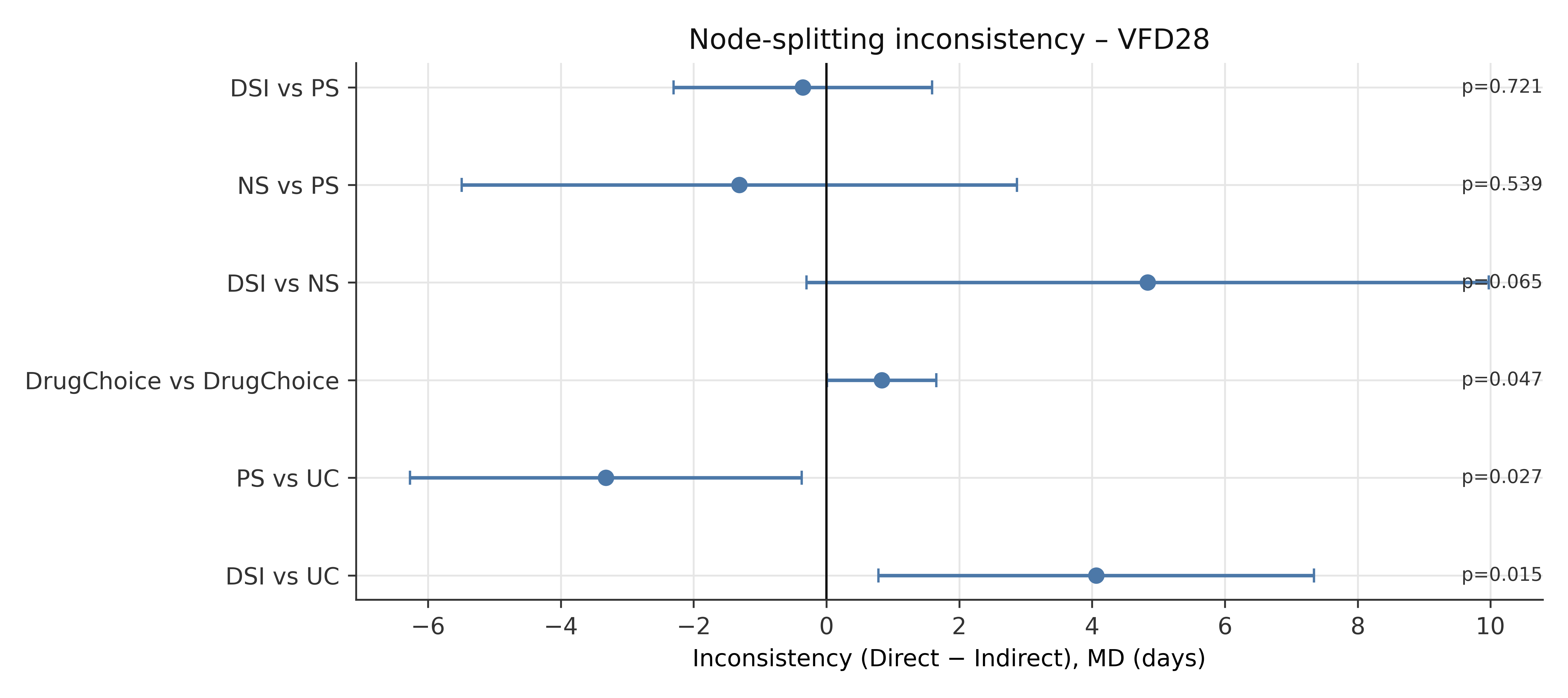


**Fig. S3. Node-splitting assessment. Local disagreement between direct and indirect evidence can be inspected here at the comparison level.**


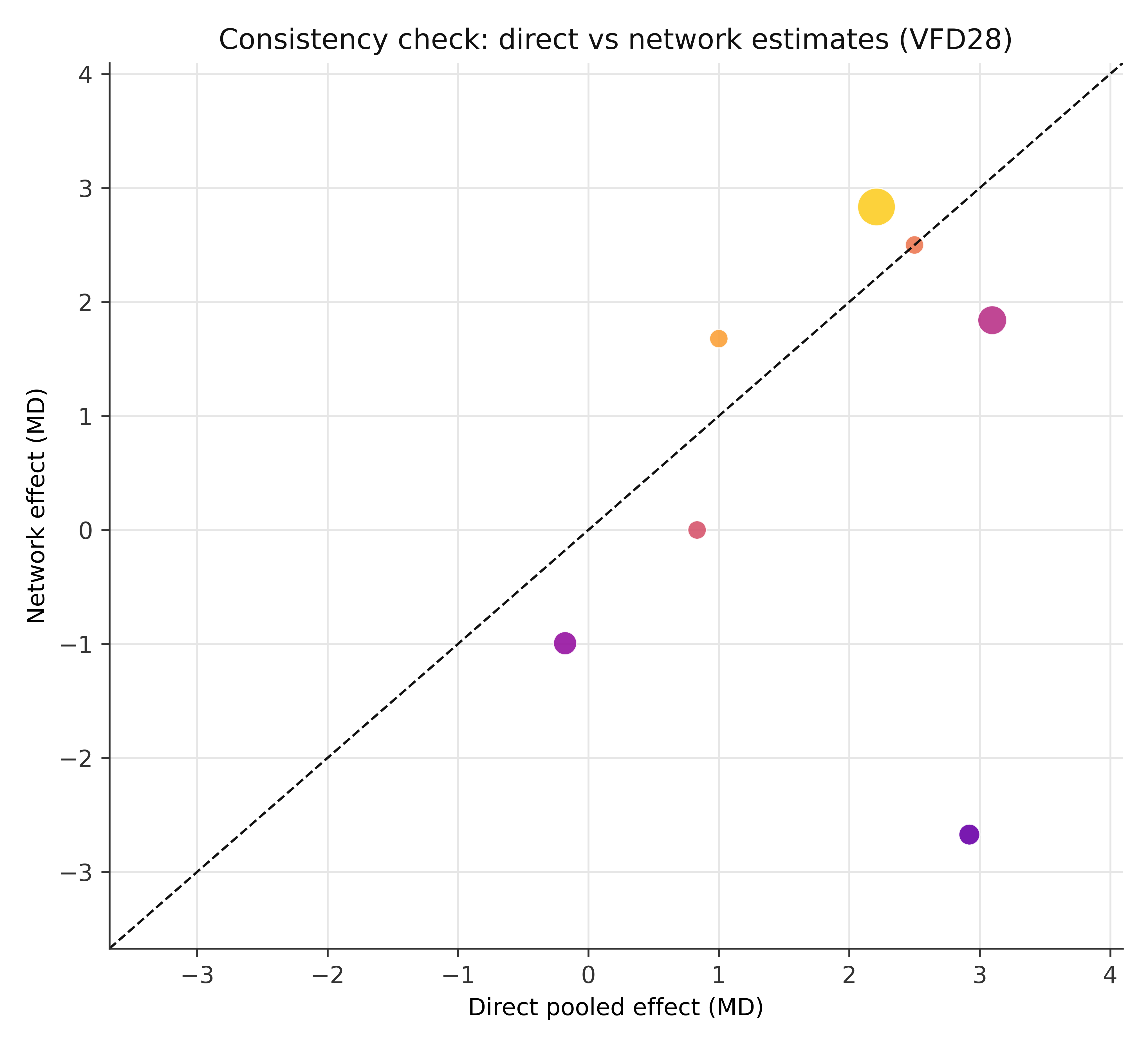


**Fig. S4. Direct versus network estimates. Agreement between the two supports the internal coherence of the main VFD28 synthesis.**


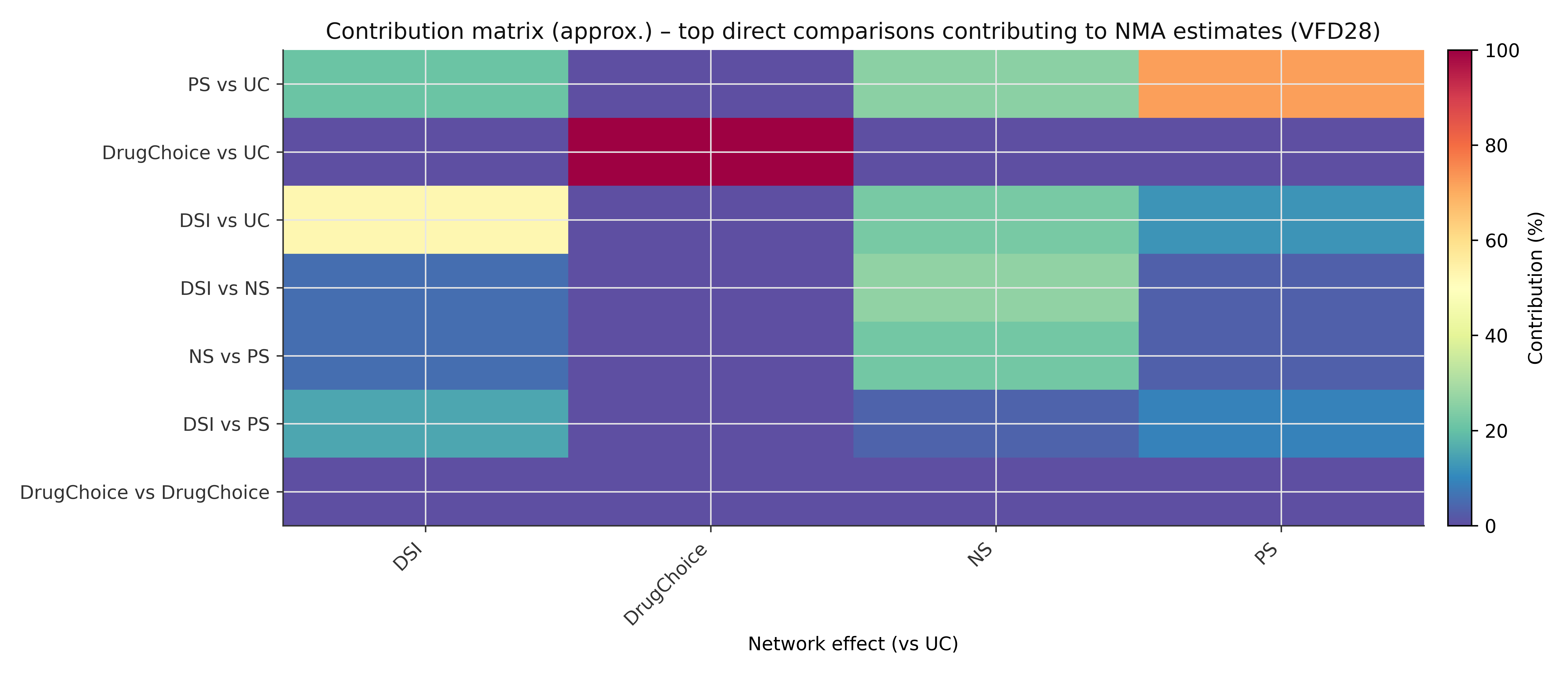


**Fig. S5. Contribution matrix. This figure shows which direct comparisons contribute most strongly to the final network estimates.**


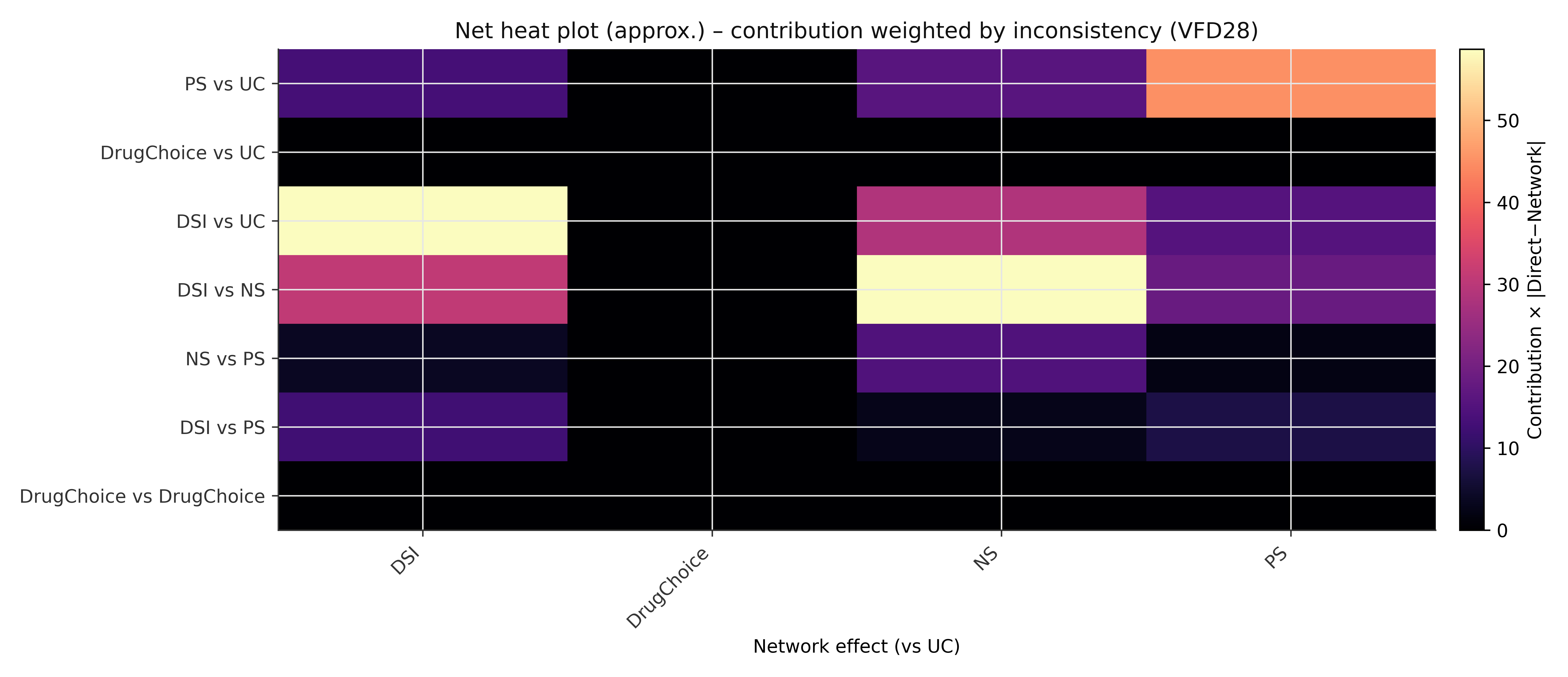


**Fig. S6. Net heat plot. Local concentration of inconsistency or heterogeneity can be localised visually in this diagnostic.**


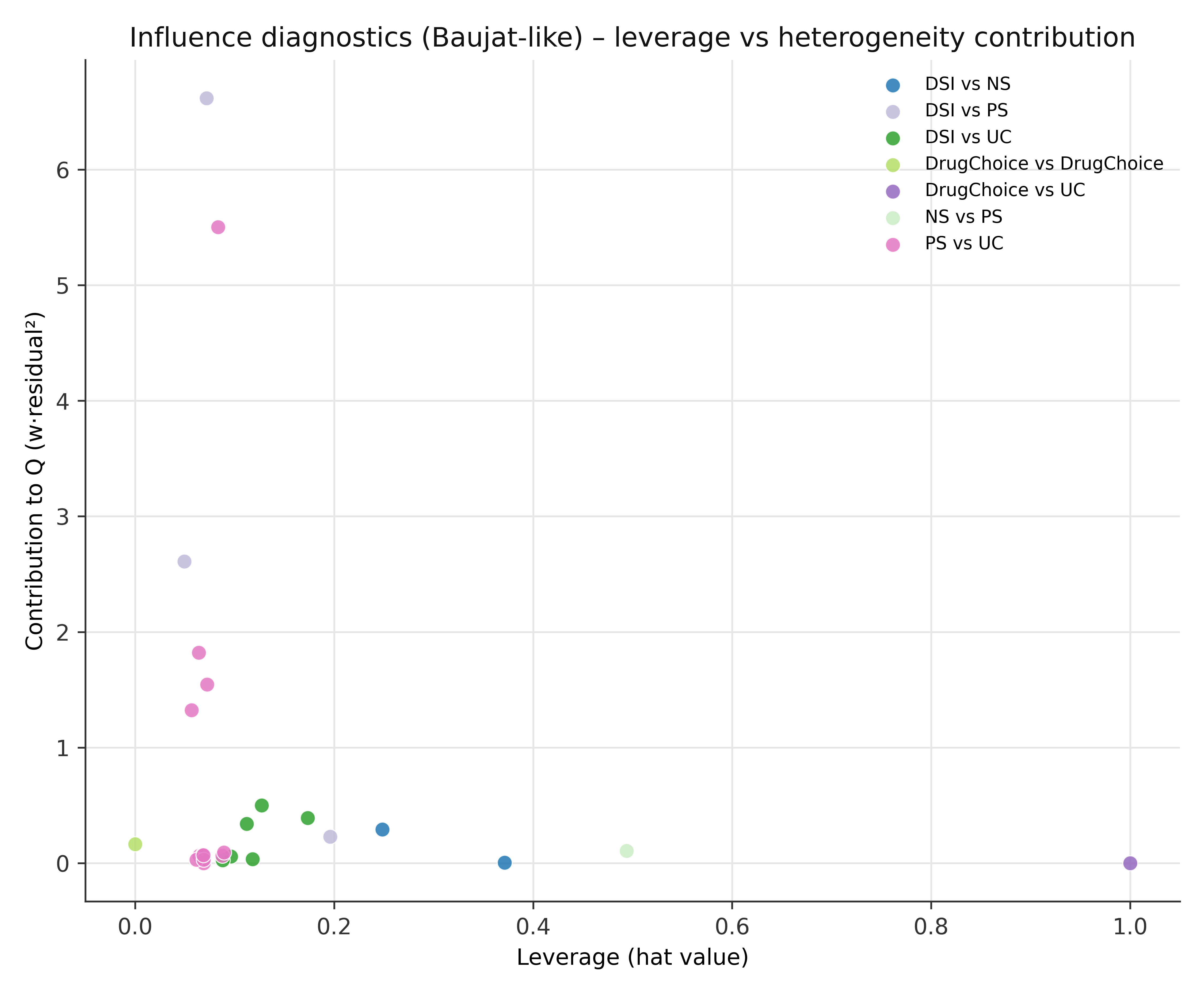


**Fig. S7. Influence diagnostics. High-leverage studies and Q contributions are displayed to show whether the network is being driven by a small number of influential trials.**


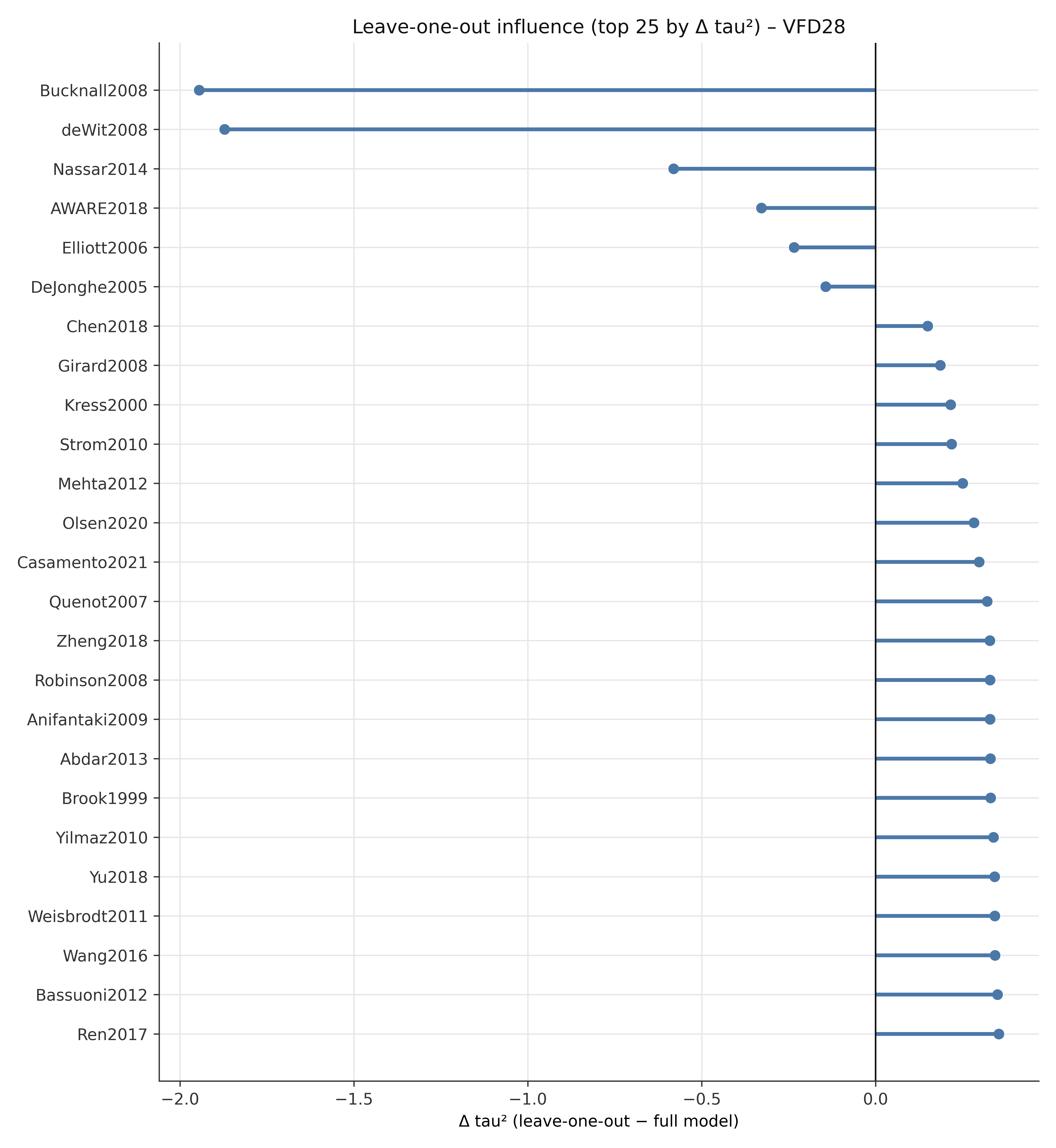


**Fig. S8. Leave-one-out change in heterogeneity. This analysis quantifies how much between-study heterogeneity changes when each study is removed in turn.**


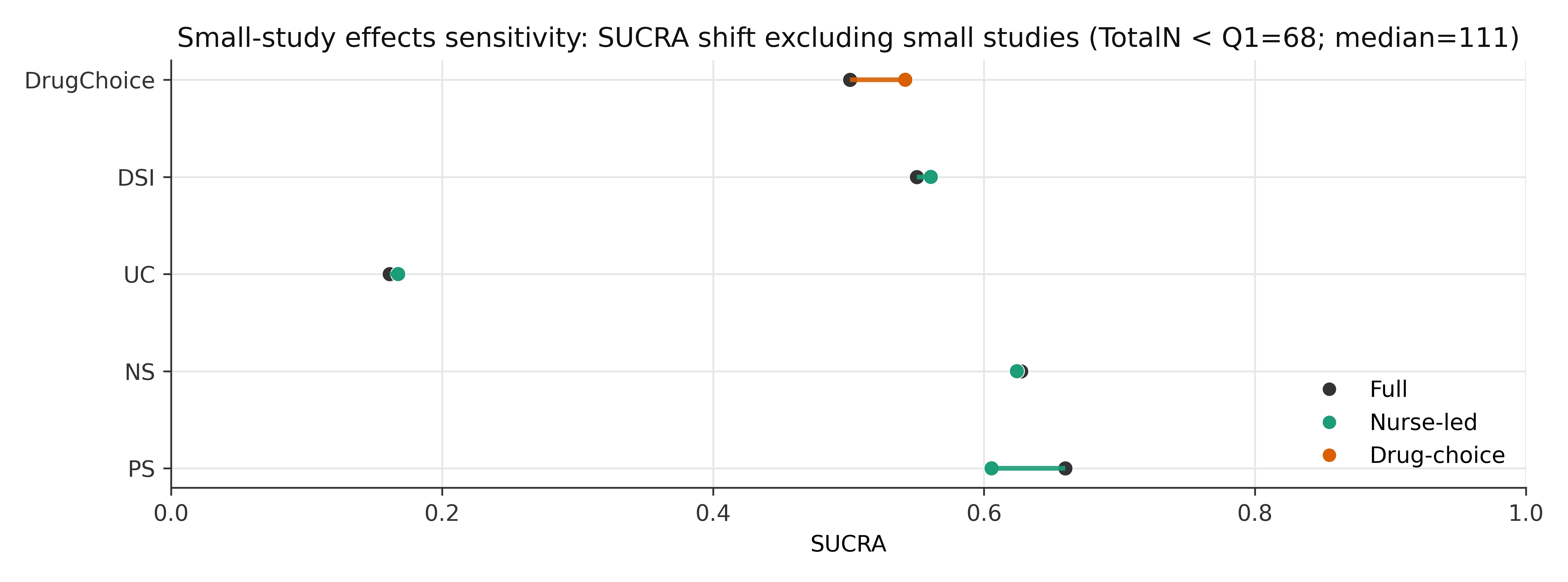


**Fig. S9. SUCRA shift after excluding smaller studies. The limited movement in rank supports stability of the main strategy-level message.**

**
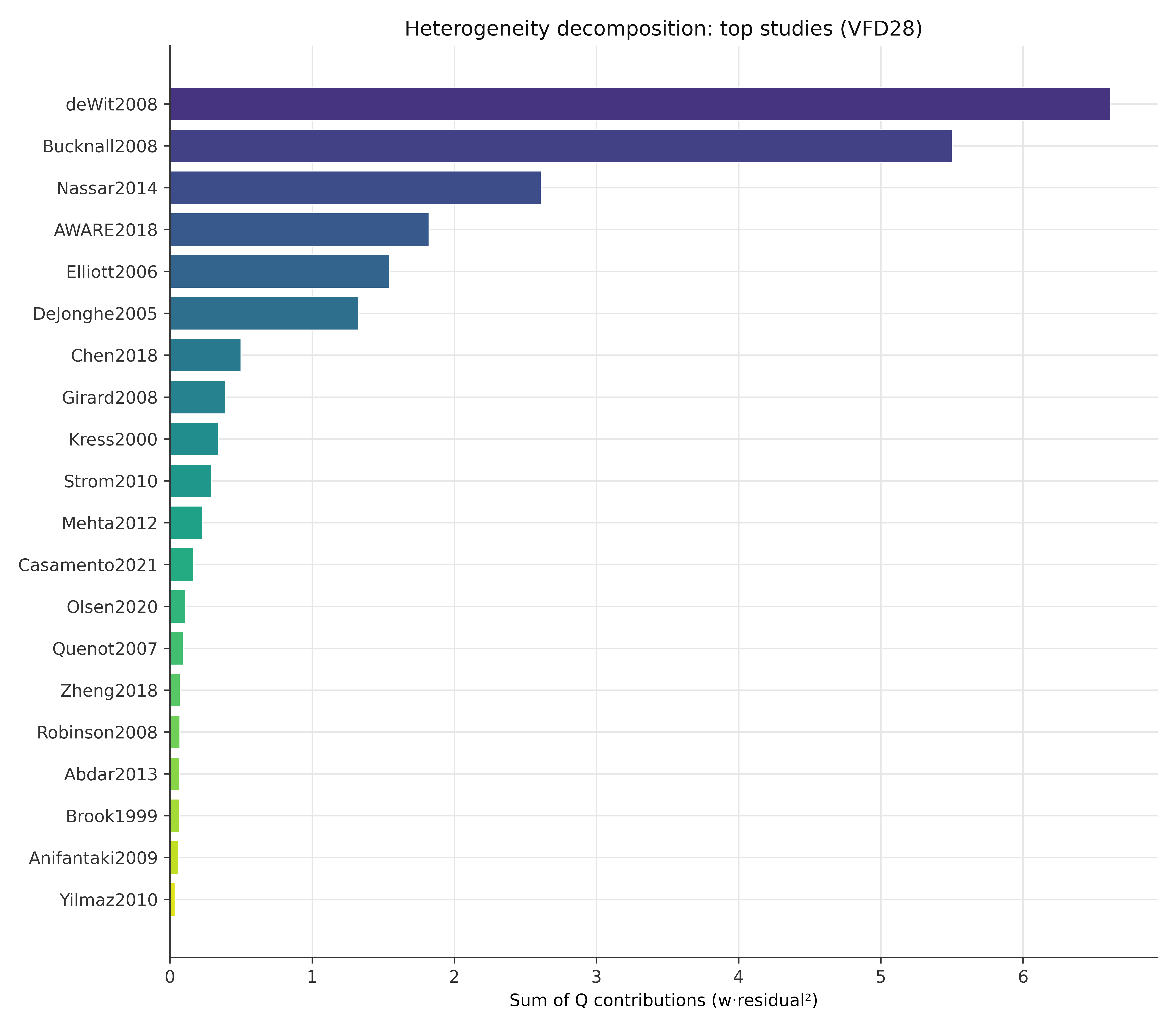
**

**Fig. S10. Heterogeneity hotspots by study.**


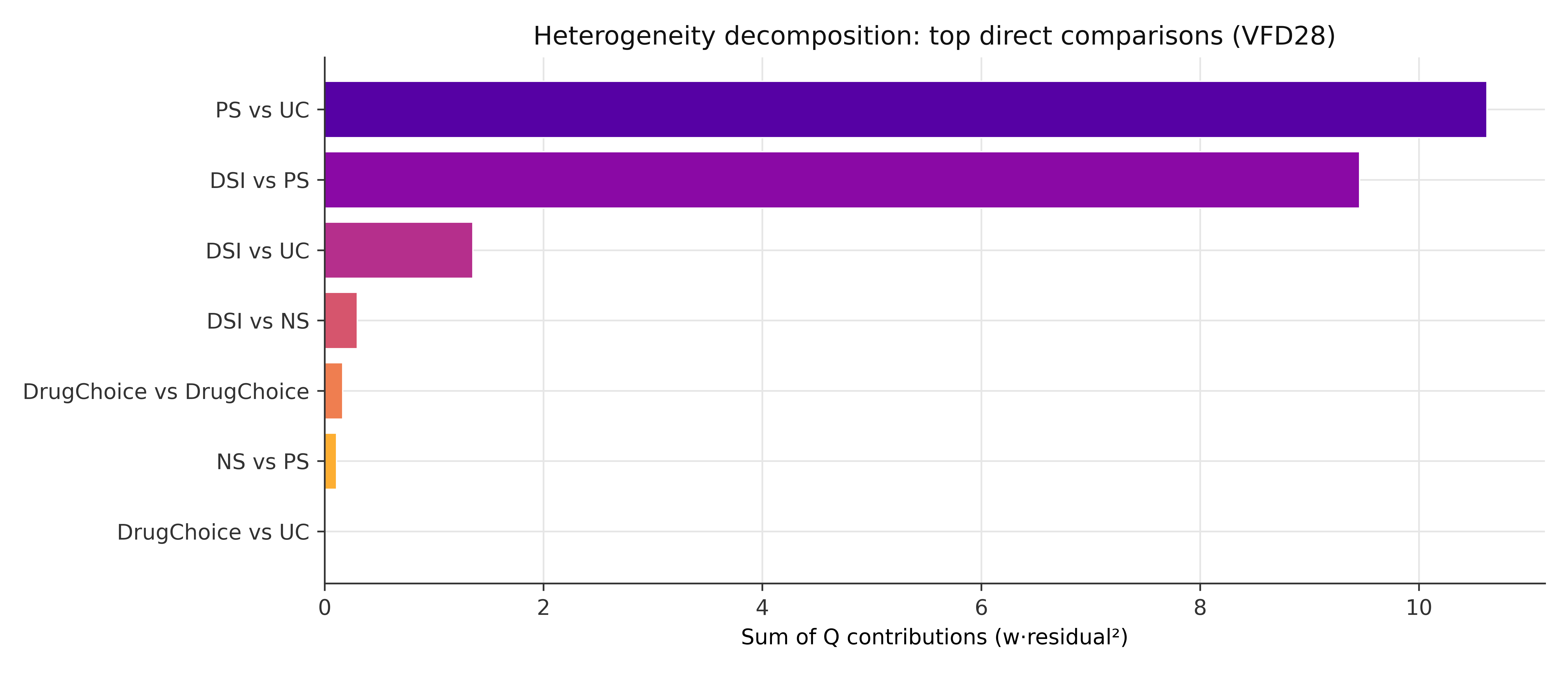


**Fig. S11. Heterogeneity hotspots by comparison. The main burden of residual heterogeneity is concentrated in a small number of specific contrasts rather than being diffuse across the whole network.**


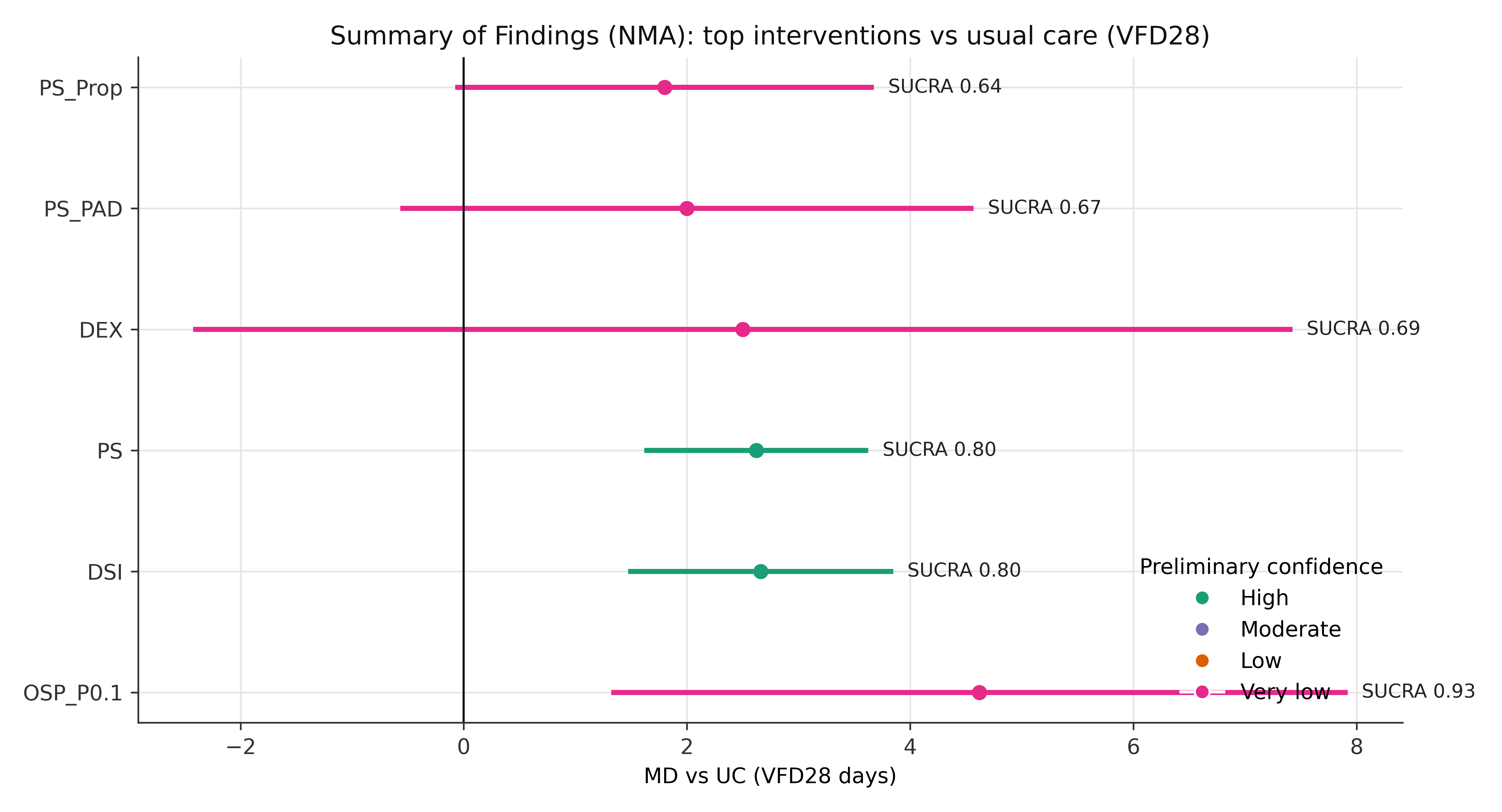


**Fig. S12. Summary-of-findings display for the leading VFD28 comparisons. This figure foregrounds why protocolised sedation and daily sedation interruption are clinically stronger conclusions than the numerically top-ranked but weakly supported node.**


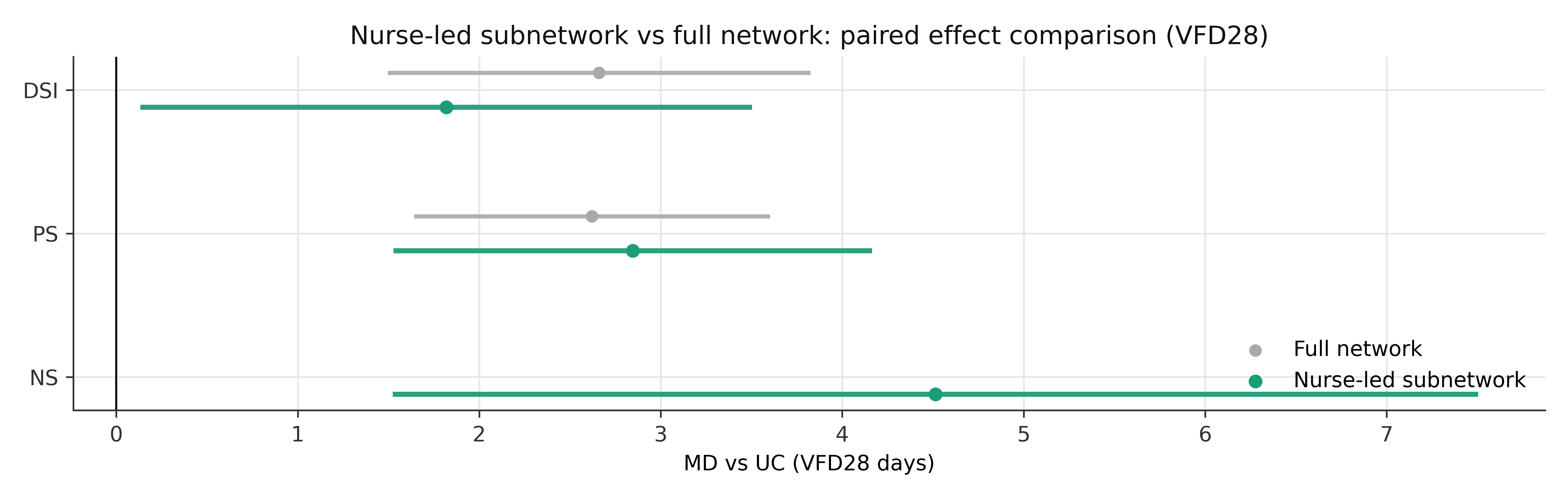


**Fig. S13. Nurse-only versus full-network paired estimates. This paired comparison demonstrates that the nursing-focused restriction preserves the direction of the main finding.**

**
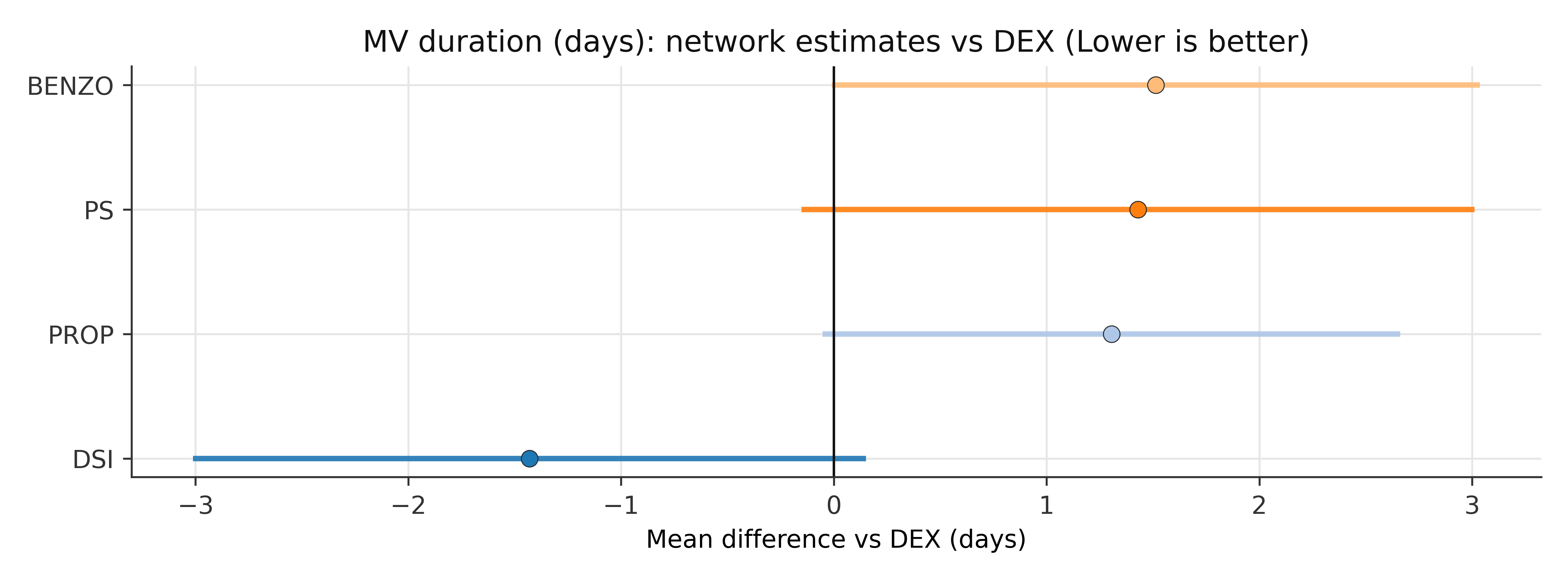
**

**Fig. S14. Secondary outcome: duration of mechanical ventilation, forest plot. These results are supportive only and should not be used to replace the VFD28 primary interpretation.**


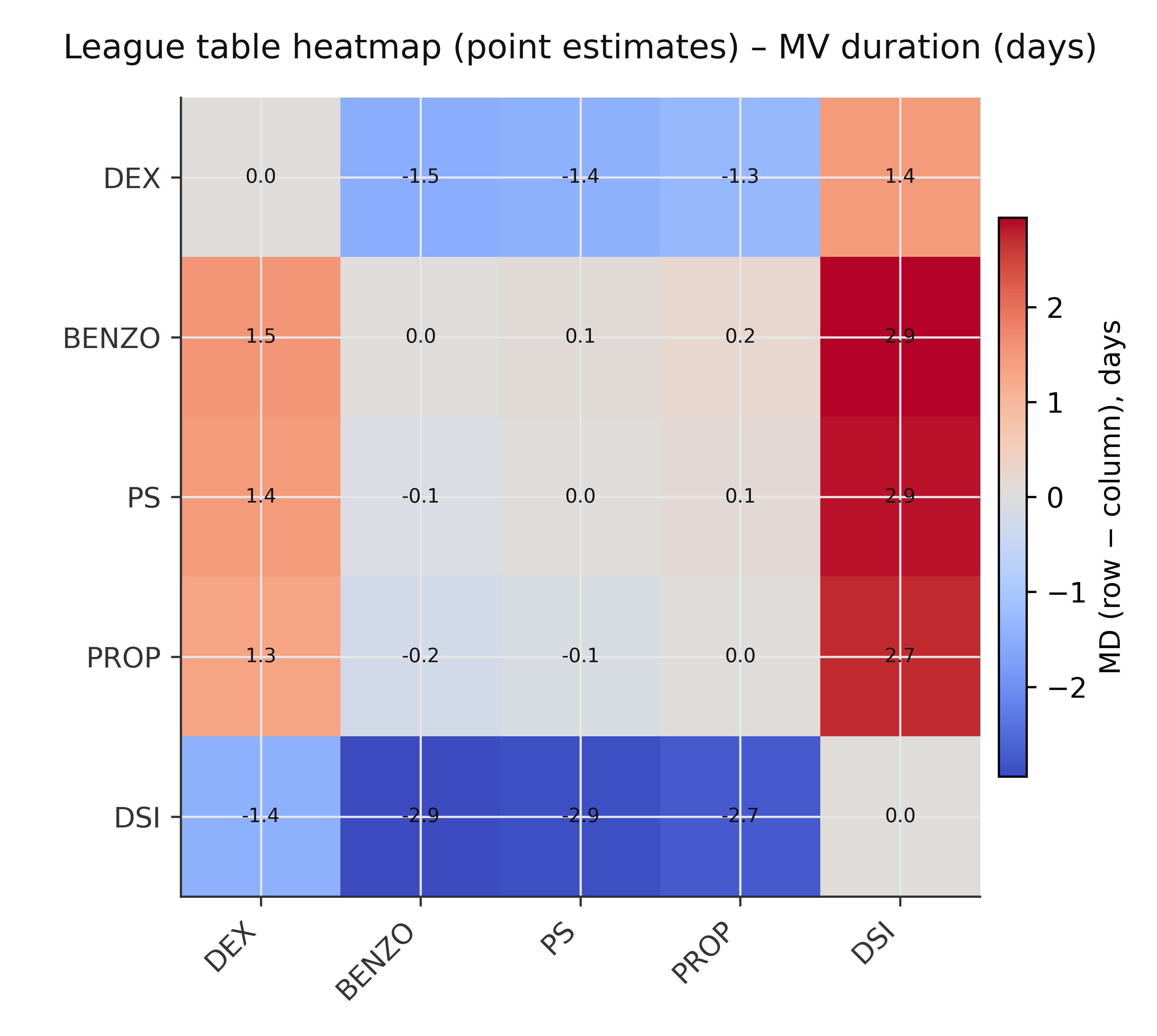


**Fig. S15. Secondary outcome: duration of mechanical ventilation, league heatmap. The subnetwork is smaller and more dependent on drug-choice pathways than the main VFD28 network.**


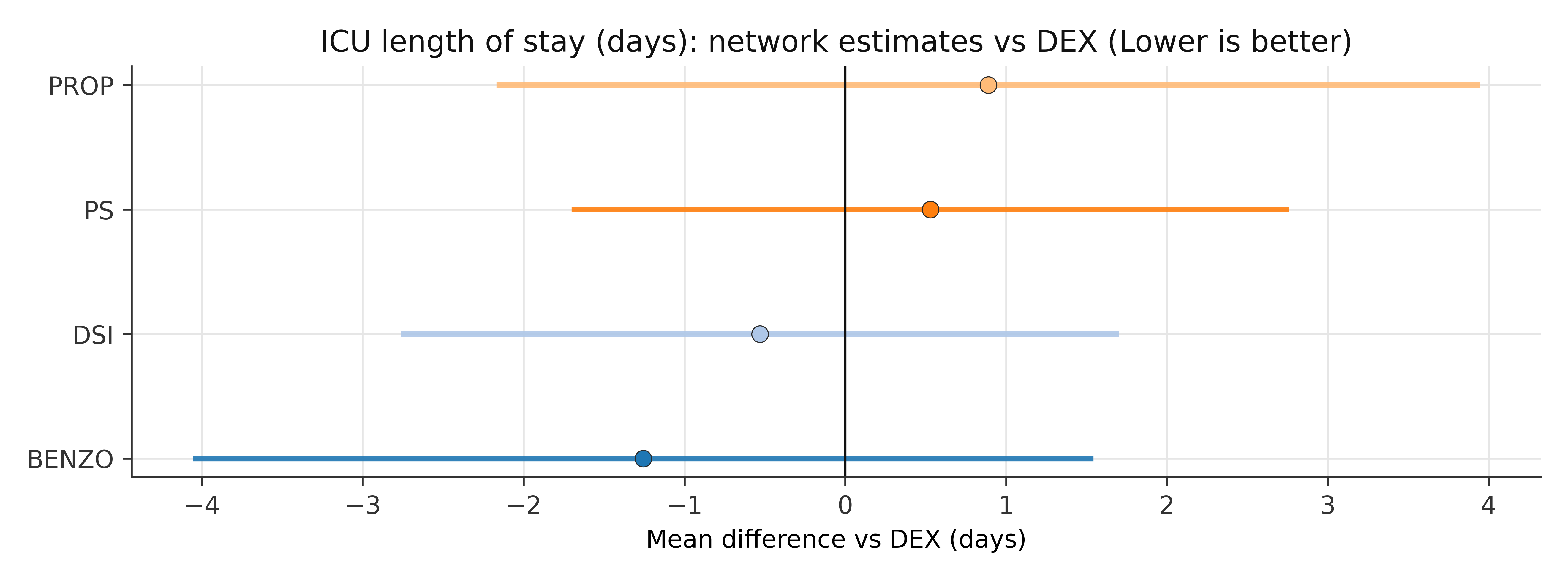


**Fig. S16. Secondary outcome: ICU length of stay, forest plot. Direction is broadly compatible with the main narrative but inferential weight is lower.**


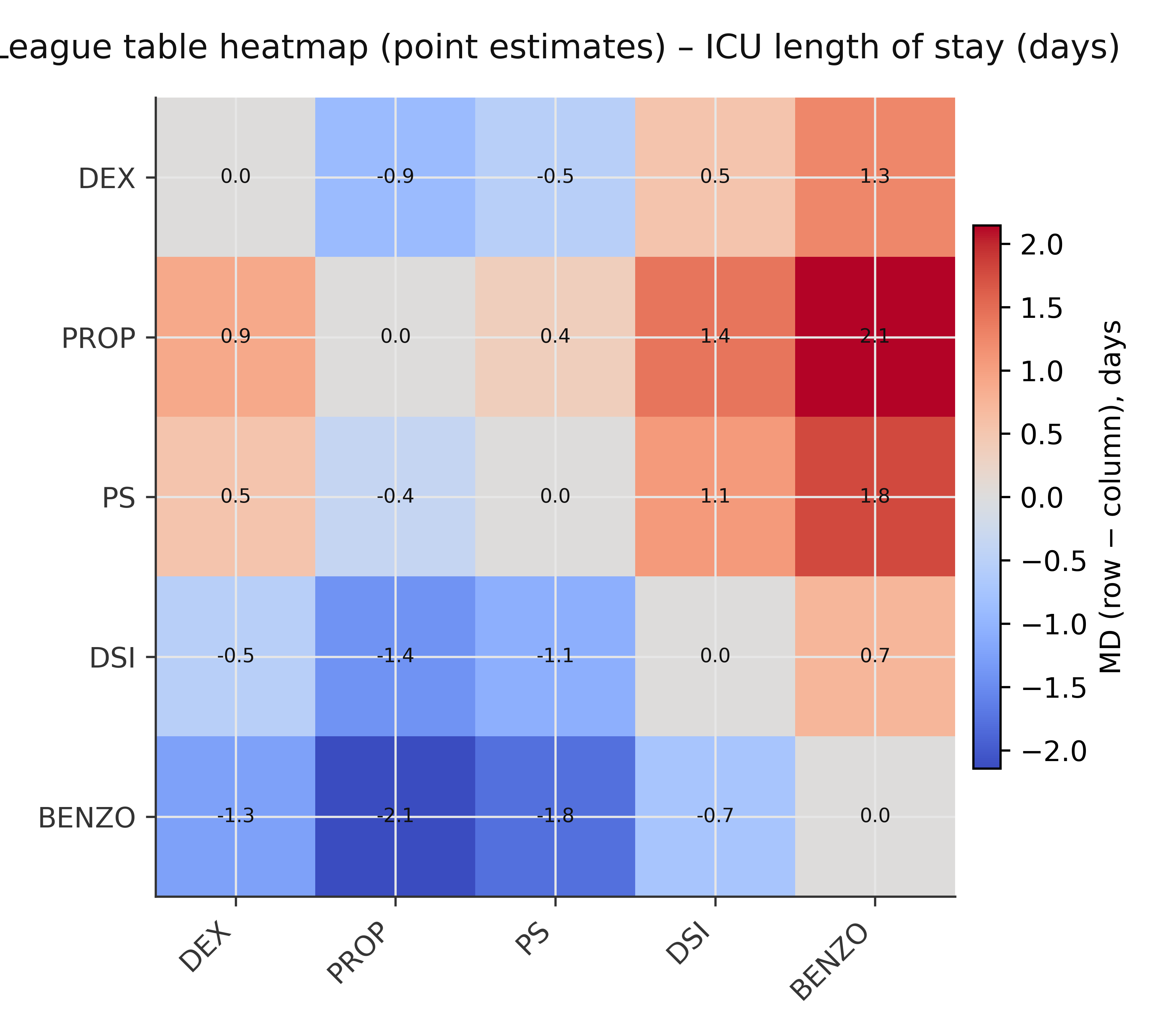


**Fig. S17. Secondary outcome: ICU length of stay, league heatmap. This figure is retained for completeness rather than for practice-defining inference.**

Supplementary references

1. Page MJ, McKenzie JE, Bossuyt PM, et al. The PRISMA 2020 statement: an updated guideline for reporting systematic reviews. BMJ. 2021;372:n71.

2. Hutton B, Salanti G, Caldwell DM, et al. The PRISMA extension statement for reporting of systematic reviews incorporating network meta-analyses of health care interventions: checklist and explanations. Ann Intern Med. 2015;162(11):777-784.

3. Sterne JAC, Savovic J, Page MJ, et al. RoB 2: a revised tool for assessing risk of bias in randomised trials. BMJ. 2019;366:l4898.

4. Nikolakopoulou A, Higgins JPT, Papakonstantinou T, et al. CINeMA: an approach for assessing confidence in the results of a network meta-analysis. PLoS Med. 2020;17(4):e1003082.

5. Barrientos-Vega R, et al. Prolonged sedation of critically ill patients with midazolam or propofol. Crit Care Med. 1997;25(6). doi:10.1097/00003246-199706000-00017. PMID:9187614.

6. Brook AD, Ahrens TS, Schaiff R, Prentice D, Sherman G, Shannon W, Kollef MH. Effect of a nursing-implemented sedation protocol on the duration of mechanical ventilation. Crit Care Med. 1999;27(12):2609-2615. doi:10.1097/00003246-199912000-00001. PMID:10628598.

7. Kress JP, Pohlman AS, O'Connor MF, Hall JB. Daily interruption of sedative infusions in critically ill patients undergoing mechanical ventilation. N Engl J Med. 2000;342(20):1471-1477. doi:10.1056/NEJM200005183422002. PMID:10816184.

8. Corbett SM, Rebuck JA, Greene CM, Callas PW, Neale BW, Healey MA, Leavitt BJ. Dexmedetomidine does not improve patient satisfaction when compared with propofol during mechanical ventilation. Crit Care Med. 2005;33(5):940-945. doi:10.1097/01.ccm.0000162565.18193.e5. PMID:15891317.

9. De Jonghe B, Bastuji-Garin S, Fangio P, et al. Sedation algorithm in critically ill patients without acute brain injury. Crit Care Med. 2005;33(1):120-127. doi:10.1097/01.CCM.0000150018.17286.79. PMID:15644658.

10. Carson SS, Kress JP, Rodgers JE, Vinayak A, Campbell-Bright S, Levitt J, et al. A randomized trial of intermittent lorazepam versus propofol with daily interruption in mechanically ventilated patients. Crit Care Med. 2006;34(5):1326-1332. doi:10.1097/01.CCM.0000215513.63207.7F. PMID:16540958.

11. Elliott R, McKinley S, Aitken LM, Hendrikz J. The adoption of a sedation scoring system and a sedation guideline in an Australian intensive care unit. J Adv Nurs. 2006. PMID:16553707.

12. Pandharipande PP, Pun BT, Herr DL, Maze M, Girard TD, Miller RR, et al. Effect of sedation with dexmedetomidine vs lorazepam on acute brain dysfunction in mechanically ventilated patients: the MENDS randomized controlled trial. JAMA. 2007;298(22):2644-2653. doi:10.1001/jama.298.22.2644. PMID:18073360.

13. Quenot JP, Ladoire S, Devoucoux F, et al. Effect of a nurse-implemented sedation protocol on the incidence of ventilator-associated pneumonia. Crit Care Med. 2007;35(9):2031-2036. doi:10.1097/01.ccm.0000282733.83089.4d. PMID:17855817.

14. Bucknall TK, Manias E, Presneill JJ. A randomized trial of protocol-directed sedation management for mechanical ventilation in an Australian intensive care unit. Crit Care Med. 2008;36(5):1444-1450. doi:10.1097/CCM.0b013e318168f82d. PMID:18434914.

15. Girard TD, Kress JP, Fuchs BD, Thomason JWW, Schweickert WD, Pun BT, et al. Efficacy and safety of a paired sedation and ventilator weaning protocol for mechanically ventilated patients in intensive care (ABC trial): a randomised controlled trial. Lancet. 2008;371(9607):126-134. doi:10.1016/S0140-6736(08)60105-1. PMID:18191684.

16. Robinson BRH, et al. An analgesia-delirium-sedation protocol for critically ill trauma patients reduces ventilator days and hospital length of stay. J Trauma. 2008. doi:10.1097/TA.0b013e318181b8f6. PMID:18784563.

17. de Wit M, Gennings C, Jenvey WI, Epstein SK. Randomized trial comparing daily interruption of sedation and nursing-implemented sedation algorithm in medical intensive care unit patients. Crit Care. 2008;12(3):R70. doi:10.1186/cc6908.

18. Anifantaki S, Prinianakis G, Vitsaksaki E, Katsouli V, Mari S, Symianakis A, et al. Daily interruption of sedative infusions in an adult medical-surgical intensive care unit: randomized controlled trial. J Adv Nurs. 2009;65(5):1054-1060. doi:10.1111/j.1365-2648.2009.04967.x. PMID:19399980.

19. Maldonado JR, Wysong A, van der Starre PJA, Block T, Miller C, Reitz BA. Dexmedetomidine and the reduction of postoperative delirium after cardiac surgery. Psychosomatics. 2009;50(3):206-217. doi:10.1176/appi.psy.50.3.206. PMID:19567759.

20. Treggiari MM, Romand J-A, Yanez ND, Deem SA, Goldberg J, Hudson L, et al. Randomized trial of light versus deep sedation on mental health after critical illness. Crit Care Med. 2009;37(9):2527-2534. doi:10.1097/CCM.0b013e3181a5689f. PMID:19602975.

21. Skrobik Y, Ahern S, Leblanc M, Marquis F, Awissi DK, Kavanagh BP. Protocolized intensive care unit management of analgesia, sedation, and delirium improves analgesia and subsyndromal delirium rates. Anesth Analg. 2010;111(2):451-463. doi:10.1213/ANE.0b013e3181d7e1b8. PMID:20375300.

22. Strøm T, Martinussen T, Toft P. A protocol of no sedation for critically ill patients receiving mechanical ventilation: a randomised trial. Lancet. 2010;375(9713):475-480. doi:10.1016/S0140-6736(09)62072-9. PMID:20116842.

23. Yilmaz M, et al. The effect of nursing-implemented sedation protocol on duration of mechanical ventilation and ICU stay: a randomized trial. Ulus Travma Acil Cerrahi Derg. 2010. PMID:21153945.

24. Weisbrodt L, McKinley S, Marshall AP, Cole L, Seppelt IM, Delaney A. Daily interruption of sedation in patients receiving mechanical ventilation. Am J Crit Care. 2011;20(4):e90-e98. doi:10.4037/ajcc2011415. PMID:21724628.

25. Bassuoni AS, et al. Patient-ventilator asynchrony during daily interruption of sedation versus no sedation protocol. Anesth Essays Res. 2012;6(2):151-156. doi:10.4103/0259-1162.108296. PMID:25885608.

26. Mehta S, Burry L, Cook D, Fergusson D, Steinberg M, Granton J, et al. Daily sedation interruption in mechanically ventilated critically ill patients cared for with a sedation protocol: a randomized controlled trial. JAMA. 2012;308(19):1985-1992.

27. Shehabi Y, et al. Early goal-directed sedation versus standard sedation in mechanically ventilated critically ill patients: a pilot study. Crit Care Med. 2013. PMID:23863230.

28. Abdar ME, et al. Effects of nurses' practice of a sedation protocol on mechanically ventilated ICU patients: a clinical trial. 2013. PMID:24403942.

29. Nassar AP Jr, Park M. Daily sedative interruption versus intermittent sedation in mechanically ventilated critically ill patients: a randomized trial. Ann Intensive Care. 2014;4:14. doi:10.1186/2110-5820-4-14.

30. Wang ZH, Sun LP, Li YW, Zhou GH. Application of continuous quality improvement in sedation management for early mechanically ventilated patients. Chinese Journal of Critical Care Intensive Care Medicine. 2018;4(1):36-41.

31. Kawazoe Y, Miyamoto K, Morimoto T, Yamamoto T, Fuke A, Hashimoto A, et al. Effect of dexmedetomidine on mortality and ventilator-free days in patients requiring mechanical ventilation with sepsis: a randomized clinical trial. JAMA. 2017;317(13):1321-1328. doi:10.1001/jama.2017.2088. PMID:28322414.

32. Ren S. Clinical effect of targeted sedative and analgesia protocol led by ICU nurses. Today Nurse. 2017;10(5):110-115.

33. Chen Q, Luo L, Xiang Z, Yin X. The application of nurse-led sedative and analgesic safety management in ICU patients on mechanical ventilation. Chinese Rural Health Service Administration. 2018;38.

34. SRLF Trial Group. Impact of oversedation prevention in ventilated critically ill patients: a randomized trial—the AWARE study. Ann Intensive Care. 2018;8(1):93. doi:10.1186/s13613-018-0425-3. PMID:30242747.

35. Yu L, Huang X, Chen Y, Wang C, Fang J, Hong D. Effect of nurse-led early sedative and analgesic management on delirium in patients on mechanical ventilation. Zhejiang Clinical Medical Journal. 2018;20.

36. Zheng Q. Clinical analysis of nurse-led sedation and analgesia protocol in severe pneumonia patients on mechanical ventilation. Journal of Clinical Medicine. 2018;5.

37. Shehabi Y, Howe BD, Bellomo R, Arabi YM, Bailey M, Bass FE, et al; ANZICS Clinical Trials Group and the SPICE III Investigators. Early sedation with dexmedetomidine in critically ill patients. N Engl J Med. 2019;380(26):2506-2517. doi:10.1056/NEJMoa1904710. PMID:31112380.

38. Olsen HT, Nedergaard HK, Strøm T, Oxlund J, Wian K-A, Ytrebø LM, et al. Nonsedation or light sedation in critically ill, mechanically ventilated patients. N Engl J Med. 2020;382(12):1103-1111. doi:10.1056/NEJMoa1906759. PMID:32068366.

39. Casamento AJ, Serpa Neto A, Young M, Lawrence M, Taplin C, Eastwood GM, et al. A Phase II cluster-crossover randomized trial of fentanyl versus morphine for analgosedation in mechanically ventilated patients. Am J Respir Crit Care Med. 2021;204(11):1286-1294. doi:10.1164/rccm.202106-1515OC. PMID:34543581.

40. Patel A, Garg R, Bharti SJ, Kumar V, Gupta N, Mishra S, Bhatnagar S, Kumar A. Comparison of sedation efficacy of intravenous infusion of dexmedetomidine versus propofol in terms of opioid consumption in patients requiring postoperative mechanical ventilation after head and neck onco-surgeries: a randomized prospective study. Indian J Cancer. 2024;61(1):81-89. doi:10.4103/ijc.IJC_949_20.

41. Hughes CG, Mailloux PT, Devlin JW, Swan JT, Sanders RD, Anzueto A, et al; MENDS2 Study Investigators. Dexmedetomidine or propofol for sedation in mechanically ventilated adults with sepsis. N Engl J Med. 2021;384(15):1424-1436. doi:10.1056/NEJMoa2024922. PMID:33528922.

42. Azuma K, Takaesu Y, Soeda H, Inagaki T, Murakami Y, Okinaga T, et al. Ability of suvorexant to prevent delirium in patients in the intensive care unit: a randomized controlled trial. Acute Med Surg. 2018;5(4):362-368. PMID:30338083.
